# Light-Inducible Spatio-Temporal Control of TLR4 and NF-κB-Gluc Reporter in Human Pancreatic Cell Line

**DOI:** 10.3390/ijms22179232

**Published:** 2021-08-26

**Authors:** Anna Stierschneider, Petra Grünstäudl, Katrin Colleselli, Josef Atzler, Christian T. Klein, Harald Hundsberger, Christoph Wiesner

**Affiliations:** 1 Department of Medical and Pharmaceutical Biotechnology, IMC University of Applied Sciences, 3500 Krems, Austria; anna.stierschneider@fh-krems.ac.at (A.S.); petra.gruenstaeudl@fh-krems.ac.at (P.G.); katrin.colleselli@fh-krems.ac.at (K.C.); christian.klein@fh-krems.ac.at (C.T.K.); harald.hundsberger@fh-krems.ac.at (H.H.); 2 Molecular Devices, LLC, 5071 Wals-Siezenheim, Austria; josef.atzler@moldev.com

**Keywords:** pancreatic adenocarcinoma, Human Toll-like receptor 4, nuclear factor kappa B, optogenetic control, attachment and invasion, invadopodia formation

## Abstract

Augmented Toll-like receptor 4 (TLR4) expression was found in nearly 70% of patients with pancreatic adenocarcinoma, which is correlated with increased tumorigenesis and progression. In this study, we engineered a new light-oxygen-voltage-sensing (LOV) domain-based optogenetic cell line (opto-TLR4 PANC-1) that enables time-resolved activation of the NF-κB and extracellular-signal regulated kinases (ERK)1/2 signalling pathway upon blue light-sensitive homodimerisation of the TLR4-LOV fusion protein. Continuous stimulation with light indicated strong p65 and ERK1/2 phosphorylation even after 24 h, whereas brief light exposure peaked at 8 h and reached the ground level 24 h post-illumination. The cell line further allows a voltage-dependent TLR4 activation, which can be continuously monitored, turned on by light or off in the dark. Using this cell line, we performed different phenotypic cell-based assays with 2D and 3D cultures, with the aim of controlling cellular activity with spatial and temporal precision. Light exposure enhanced cell attachment, the formation and extension of invadopodia, and cell migration in 3D spheroid cultures, but no significant changes in proliferation or viability could be detected. We conclude that the opto-TLR4 PANC-1 cell line is an ideal tool for investigating the underlying molecular mechanisms of TLR4, thereby providing strategies for new therapeutic options.

## 1. Introduction

Pancreatic adenocarcinoma is a devastating malignancy and accounts for 4.5% of all deaths caused by cancer worldwide, making it the seventh leading cause of global cancer deaths. The 5-year survival rate for people with pancreatic cancer is about 8% on average and depends on whether the cancer is detected at an early (32%) or late stage (3%) [1]. Pancreatic adenocarcinoma is one of the greatest challenges in oncology due to the late diagnosis and the lack of curative therapeutic strategies [2]. Toll-like receptors (TLRs) promote pancreatic carcinogenesis and epithelial-mesenchymal transition and, therefore, represent a promising target class for cancer therapy [3,4]. TLRs are a family of evolutionarily conserved pattern-recognition receptors (PRR) that play a key role in the innate immune response and largely determine the development of the adaptive immune response. TLRs are class-type I transmembrane proteins composed of an extracellular domain (ectodomain), a transmembrane region, and an intracellular domain. The ectodomain consists of leucine-rich repeats and two to four evolutionarily conserved cysteine residues, which recognize and bind to evolutionarily conserved molecular motifs in pathogen-associated molecular patterns (PAMPs) of microorganisms or damage-associated molecular patterns (DAMPs) of damaged tissues to activate an innate and adaptive immune response [4,5,6].

To date, there are 11 members of the TLR family identified, of which TLR1, TLR2, TLR4, TLR5, TLR6 and TLR11 are located on the cell surface, and TLR3, TLR7, TLR8 and TLR9 are localised to the endosomal/lysosomal compartment [7]. Regarding pancreatic adenocarcinoma, recent studies show that 69% of patients with pancreatic cancer display an elevated expression of TLR4, which is correlated with an increased activation of the nuclear factor kappa B (NF-κB), phosphoinositol-3 kinase/AKT and mitogen-associated protein kinase signalling pathways, all being involved in tumourigenesis and tumour progression [7,8]. For example, the canonical NF-κB signalling pathway regulates the expression of genes involved in angiogenesis, metastasis, cell survival, cell proliferation, inflammatory processes, and immune modulation. Therefore, diminishment or even blockage of TLR4-related pathways such as the NF-κB signalling pathway are promising strategies for possible targeted therapy.

Activation or inactivation of TLR4 by using agonists, antagonists, activators and/or inhibitors possess only limited spatial and temporal control of the underlying signalling pathway and lack the ability to either continuously change or terminate signalling [9,10,11,12]. Optogenetics is an innovative technique that makes use of genetic and optical methods to control molecular processes, cellular signals and animal behaviours spatially and temporally. In optogenetics, light-sensitive protein domains of microbial or plant photoreceptors that are capable of light-controlled inter- or intramolecular interactions are integrated into effector proteins. Consequently, light induction allows activation, inactivation, localisation, or stabilisation/destabilisation of signalling pathways, depending on the protein type and set-up [13,14]. The currently available domain repertoire allows for blue- [15,16,17], red- [18,19], or green- [20] light-induced formation of protein complexes.

Here, we engineered a novel optogenetic controllable cell line (opto-TLR4 PANC-1) in which the TLR4 can be switched on by blue light and off in the dark by fusing the light-oxygen-voltage sensing (LOV) domain to the TLR4.

PANC-1 is a human pancreatic cancer cell line isolated from a pancreatic carcinoma of ductal origin. It is characterized with a high metastatic potential and poor differentiation abilities. Above all, it is known as a suitable transfection host [21,22]. The LOV domain, isolated from the *Vaucheria frigida aureochrome 1* (yellow-green algae), noncovalently binds a flavin chromophore, which can be stimulated by blue light (λmax ≈ 470 nm) absorption to initiate a photochemical reaction that results in the formation of a covalent adduct between a conserved cysteine and the flavin ring [23]. This results in a change in protein conformation allowing for dimerisation of the LOV domains [24,25,26,27] and activation of the TLR4-LOV fusion protein, a process fully reversible in the dark. TLR4 activation and real-time detection of the underlying signalling pathways can be confirmed with the stable integrated NF-κB-Gluc reporter system. This opto-TLR4 PANC-1 reporter cell line offers a novel tool for analysing TLR4 signalling pathway and the concomitant genotypic and phenotypic effects in a time-resolved manner, and enables a new high-content analysis (HCA) approach for the screening of TLR4 signalling modulators.

## 2. Results

### 2.1. Design of a Light Inducible TLR4 Pancreatic Cell Line with Stable NF-κB-Gluc Reporter

Previously, blue light-inducible activation of several members of the receptor tyrosine kinases (Opto-RTKs) have been successfully generated by fusing light-sensitive protein domains to the far C-terminus of the RTK [15]. In this study, a similar strategy was initially employed to generate a pancreatic cell line where the photosensitive protein domain (LOV) was fused to the C-terminal end of the Toll-like receptor 4 (TLR4) to allow a spatially and temporally precise dimerisation of the TLR4 and activation of the signalling cascades.

**Figure 1:**
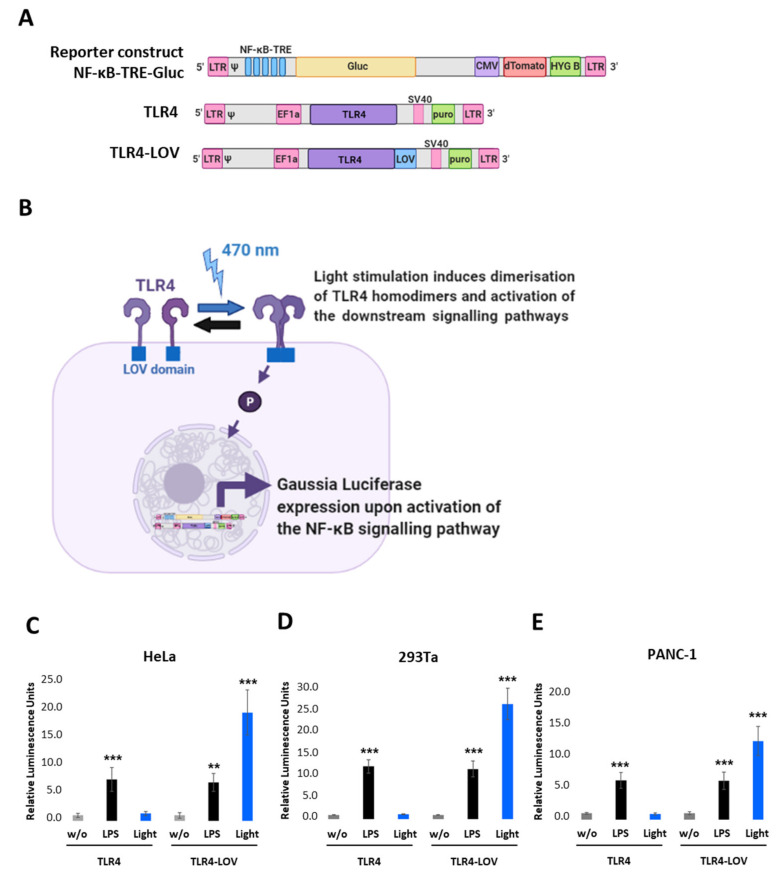
Design and functional analysis of the NF-κB-TRE-Gluc reporter and TLR4-LOV construct. (**A**) Schematic representation of the engineered NF-κB-TRE-Gluc reporter, the TLR4, and the TLR4-LOV constructs. NF-κB-TRE-Gluc encompasses five tandem repeats (TRE), Gaussia Luciferase (Gluc), dTomato under the control of CMV promoter, and Hygromycin B (HYG B). TLR4-LOV consists of a full-length human TLR4 molecule with an incorporated LOV domain at the C-terminal intracellular domain. (**B**) Schematic representation of the function of TLR4-LOV. Homodimerisation and activation of NF-κB in response to blue light can be detected in real-time via measuring Gluc. (**C**) HeLa, (**D**) 293Ta, and (**E**) PANC-1 were transiently co-transfected with either TLR4 or TLR4-LOV with the NF-κB-TRE-Gluc reporter, and Gluc was measured 6 h post LPS (100 ng/mL) or light (470 nm, 7 V, 7.5 min) treatment, respectively, or left untreated (w/o). Post-ANOVA multiple comparisons relative to the control were performed using Dunnett’s test. Error bar = SD. *n* = 6; ** *p* < 0.01, *** *p* < 0.001.

We first designed a lentiviral reporter construct with five tandem repeats of the NF- κB transcription responsive elements (NF-κB-TRE), Gaussia Luciferase (Gluc), CMV-dTomato, Hygromycin B (HYG B), and a full-length TLR4 (TLR4 in pEZ-Lv195 vector) where the cDNA of the LOV domain was inserted in-frame at the 3′ end of TLR4 (Figure 1A and Figure S1). Cells expressing these constructs should respond to blue light and the respective endogenous downstream signalling of NF-κB and should be quantifiable by measuring the Gaussia Luciferase (Figure 1B). To verify this assumption, we transiently co-transfected either TLR4 or TLR4-LOV with the NF-κB-TRE-Gluc reporter into HeLa, 293Ta, and PANC-1 and measured Gluc activity 6 h post LPS (100 ng/mL) or light (470 nm, 7 V, 7.5 min) treatment, respectively, or left untreated (w/o). As depicted in Figure 1C–E, light exposure induced high NF-κB activation in cells transfected with TLR4-LOV, but not in cells with TLR4. In comparison, LPS stimulated NF-κB activation in both TLR4 and TLR4-LOV transfected cell lines.

**Figure 2:**
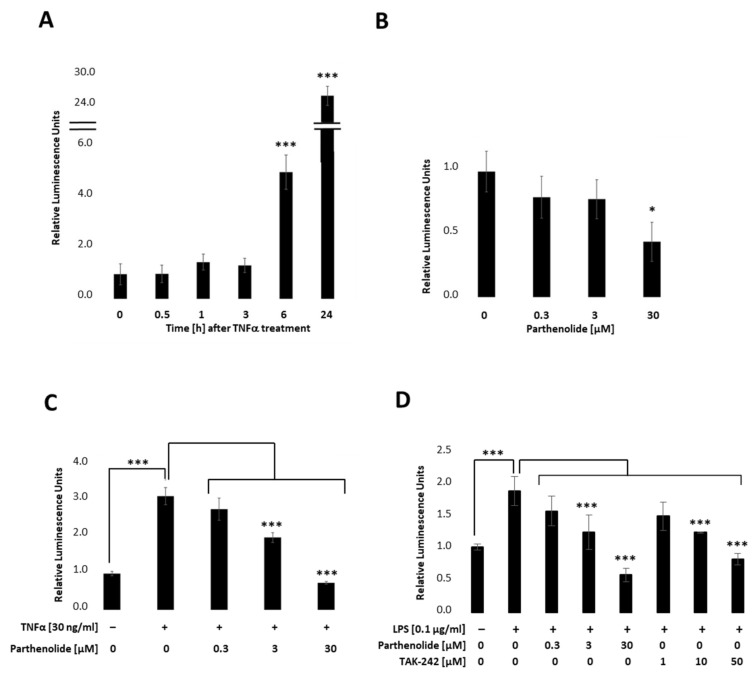
Relative NF-κB promoter-linked Gaussian Luciferase activity in PANC-1 NF-κB cells. PANC-1 NF-κB cells were stimulated with (**A**) 30 ng/mL TNFα, (**B**) 0.3 µM, 3 µM, or 30 µM PAR, or co-treated with TNFα and PAR and luciferase measured after **(A**) 0, 0.5, 1, 3, 6, and 24 h, or (**B**,**C**) 24 h. (**D**) PANC-1 NF-κB cells were non-treated, treated with 100 ng/mL LPS +/− different concentrations of PAR (0.3, 3, 30 µM) or TAK-242 (1, 10, 50 µM) and luciferase was measured after 6 h. Post-ANOVA, multiple comparisons relative to the control were performed using Dunnett’s test. Error bar = SD. *n* = 6; * *p* < 0.05; *** *p* < 0.001.

To establish a stable PANC-1 cell line that allows real-time detection we first integrated the NF-κB-TRE-Gluc reporter using the lentiviral delivery system. Transfected cells were selected by using Hygromycin B and dTomato, and the obtained cell clones picked and tested by measuring Gluc. Figure 2 indicates relative stimulation and repression for a representative PANC-1 NF-κB cell clone. Therefore, PANC-1 NF-κB cell lines (Figure 1A) were treated with 30 ng/mL TNFα, 0.3 µM, 3 µM or 30 µM PAR (Parthenolide; NF- κB inhibitor) (Figure 2B), or a combination of both (Figure 2C). Respective endogenous downstream signalling of NF-κB was quantified by measuring the Gluc reporter 24 h post-treatment by applying the BioLux^®^ Gaussia Luciferase Assay Kit. As depicted in Figure 2A, Gaussia Luciferase was increased 5-fold after 6 h and 25-fold after 24 h TNFα (30 ng/mL) treatment, whereas PAR (0.3 µM, 3 µM or 30 µM), a known NF-κB inhibitor, showed a concentration-dependent decrease in NF-κB-Gluc (Figure 2B). Combinational treatments of TNFα (30 ng/mL) and PAR (0.3 µM, 3 µM or 30 µM) showed a concentration-dependent decrease in the TNFα-stimulated luciferase activity (Figure 2C). To investigate whether the reporter cell line can also be activated by TLR4 signalling, cells were stimulated with 100 ng/mL LPS in combination with different PAR (0–30 µM) or TAK-242 (resatorvid; TLR4 inhibitor; 0–50 µM) concentrations and luciferase measured 6 h later (Figure 2D). These results clearly demonstrate that the selected PANC-1 NF-κB clone allows both up- and down-regulation of the stably integrated NF-κB-dependent Gluc reporter in a concentration-dependent manner.

**Figure 3:**
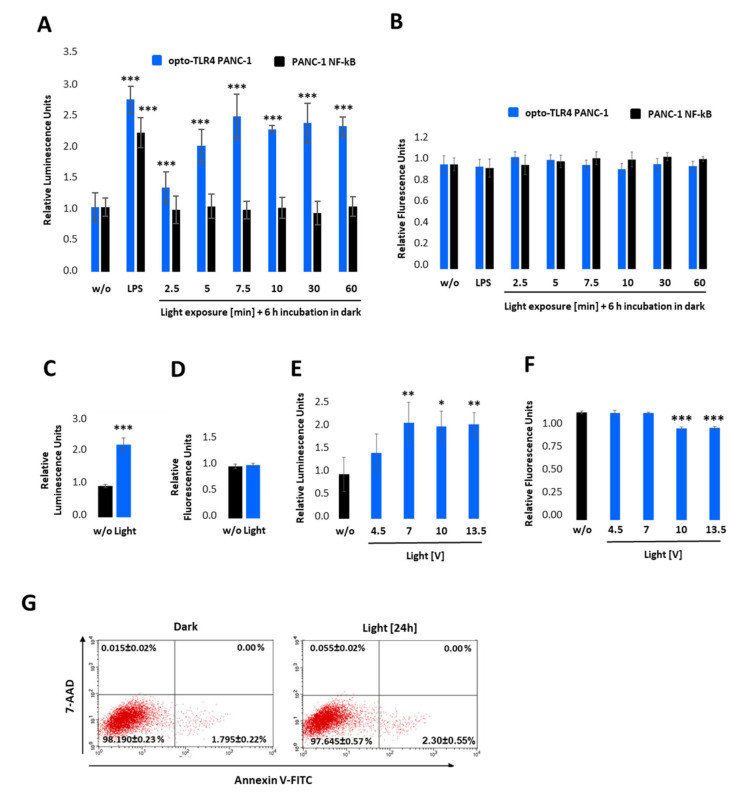
Characterisation of the opto-TLR4 PANC-1 cell line. TLR4-LOV homodimerisation and activation of NF-κB in response to blue light can be detected in real-time via measuring Gaussia Luciferase (Gluc) activity. (**A**,**B**) Opto-TLR4 PANC-1 and PANC-1 NF-κB were stimulated with 100 ng/mL LPS or exposed with light at 470 nm for 0, 2.5, 5.0, 7.5, 10, 30, or 60 min using the Amuza LED Array System, followed by incubation in the dark. After 6 h, (**A**) Gluc activity and (**B**) cell viability (PrestoBlue) were determined. (**C**,**D**) Opto-TLR4 PANC-1 cells were stimulated with light (470 nm) for 12 h, followed by (**C**) Gluc detection and (**D**) PrestoBlue. (**E**,**F**) Cells were illuminated with 4.5, 7.0, 10, and 13 V at 470 nm for 7.5 min, followed by incubation in the dark for 6 h and (**E**) relative NF-κB promoter-linked Gluc and (**F**) PrestoBlue measured. Post-ANOVA multiple comparisons relative to the control were performed using Dunnett’s test. Error bar = SD. *n* = 6; * *p* < 0.05; ** *p* < 0.01, *** *p* < 0.001. (**G**) To measure cellular toxicity after light exposure, opto-TLR4 PANC-1 cell line was exposed to light (7 V, 470 nm) for 24 h, and flow cytometry analysis performed after Annexin V-Alexa Fluor 488 and 7-AAD staining. Values are means ± standard error (*n* = 3).

Next, the engineered TLR4-LOV plasmid was stably integrated into the NF-κB PANC-1 cell line, and clones were picked and selected for TLR4-LOV expression and their ability to activate NF-κB after light stimulation at 470 nm effectively. (Figure 3 and Figure S2).

### 2.2. Light Induces TLR4-Dependent NF-κB and MAP Kinase Pathway Activation in Opto-TLR4 PANC-1 Cell Line

To test the light-inducible TLR4-LOV dimerisation/activation and subsequent signal transduction (NF-κB-Gluc), the selected clone, opto-TLR4 PANC-1, was pre-incubated overnight in serum-free culture medium and exposed to light at 470 nm for 0–60 min using the Amuza LED Array System, followed by incubation in the dark (Figure 3A). After 6 h, NF-κB signalling was determined by measuring Gluc. As depicted in Figure 3A, light exposure of opto-TLR4 PANC-1 at 470 nm and 7 V indicated a time-dependent Gluc activity peaking after 7.5 min (2.5-fold). Importantly, only opto-TLR4 PANC-1 showed Gluc activity after light exposure compared to PANC-1 NF-κB (reporter), but both cell lines could be stimulated with LPS (Figure 3A). Additionally, no change in cell viability (PrestoBlue) could be observed (Figure 3B). To investigate whether prolonged exposure or the change in voltage has an impact on TLR4-LOV/ NF-κB-Gluc reporter activity and cell viability, cells were continuously illuminated at 470 nm for 12 h at 7 V (Figure 3C,D) or stimulated for 7.5 min at 4.5-, 7-, 10- and 13-volts, followed by incubation for 6 h in the dark (Figure 3E,F). Subsequently, promoter activity and cell viability were measured. Interestingly, prolonged light exposure (12 h) or increased voltage (10 and 13.5 V) did not boost reporter activity compared to 7 V and 7.5 min; however, light intensity at lower voltage (4.5 V) indicated reduced reporter activity. In contrast, an applied voltage higher than 7 V already diminished cell viability significantly after 6 h. This observed effect is most likely due to accumulating heat and the factor of time (Figure 3F). To exclude that light at 7 V impairs cell viability, cells were exposed to light for 24 h and apoptosis and necrosis of cells were measured using annexin V and 7-AAD. No significant toxicity was detected (Figure 3G).

**Figure 4:**
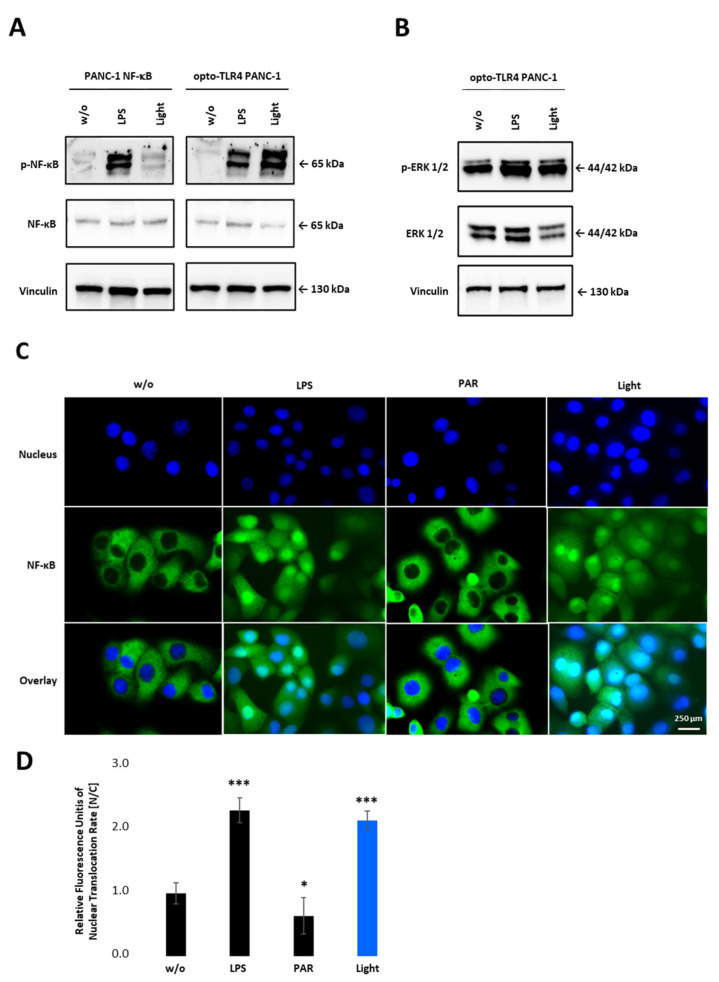
NF-κB activation upon illumination of opto-TLR4 PANC-1. (**A**) Western blot analysis with antibodies against phospho-p65, p65 and (**B**) phosphor-ERK1/2 and ERK1/2 30 min after light or LPS (100 ng/mL) stimulation. Vinculin was used as the reference gene. (**C**) Nuclear localisation of p65 after LPS (100 ng/mL) or light (470 nm, 7.5 min) stimulation compared to untreated cells or cells treated with PAR (3 µM). (**D**) Relative Fluorescence units of nuclear localisation of p65 [nucleus/cytoplasm]. Post-ANOVA multiple comparisons relative to the control were performed using Dunnett’s test. Error bar = SD. *n* = 5; * *p* < 0.05; *** *p* < 0.001.

To confirm NF-κB activity after light stimulation, phosphorylation of p65 in PANC-1 NF-κB and opto-TLR4 PANC-1 cells was evaluated. As shown in Figure 4A, light stimulation at 470 nm for 7.5 min, and subsequent incubation in the dark for 20 min, significantly increased p65 phosphorylation on Serine 529 in opto-TLR4 PANC-1 cells, but not in PANC-1 NF-κB cells. In contrast, LPS treatment (100 ng/mL) for 30 min induced p65 phosphorylation in both cell lines. Additionally, phosphorylation of ERK was only marginally increased after light or LPS stimulation compared to negative control in opto-TLR4 PANC-1 cells (Figure 4B). When measuring nuclear localisation of stained p65 (nucleus/cytoplasm), light (470 nm, 7.5 min) or LPS (100 ng/mL) induced p65 translocation two-fold compared to untreated cells, whereas NF-κB inhibition with Parthenolide (3 µM) reduced p65 localisation of 40% (Figure 4C,D).

### 2.3. TLR4 Activation Can Be Reversibly Turned on or Off by Light Illumination or Incubation in the Dark in the Opto-TLR4 PANC-1 Cell Line

**Figure 5:**
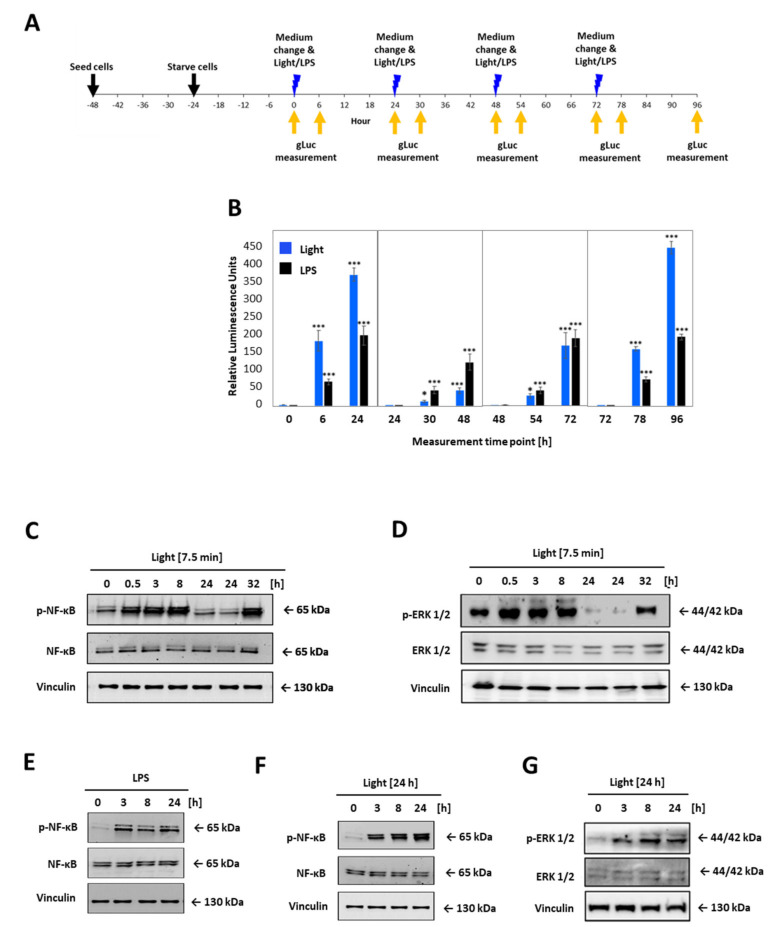
Comparative time-curve and re-activation analysis of opto-TLR4 PANC-1 cell line. Cells were exposed to light at 470 nm for 7.5 min or treated with 100 ng/mL LPS and incubated for 24 h in the dark before cells were again stimulated with light or LPS, respectively. Gluc was measured 6 h and 24 h after each stimulation. (**A**) Timeline for opto-TLR4 PANC-1 cell seeding, treatment, and imaging. (**B**) Gluc activity. Western blots of whole-cell extracts of opto-TLR4 PANC-1 cell line after light exposure (470 nm) for 7.5 min at timepoints 0 and 24 prior to extraction of proteins. Blots were probed with antibodies against (**C**) phospho-p65, total p65, or (**D**) p-ERK1/2, ERK1/2, and vinculin as loading control. Cells were treated continuously with LPS (100 ng/mL) or with light (470 nm) for 24 h and cell extracts were blotted and probed with antibodies against (**E**,**F**) phosphor-p65 p65, and (**G**) phospho-ERK1/2, ERK1/2, and vinculin as loading control. Post-ANOVA multiple comparisons relative to the control were performed using Dunnett’s test. Error bar = SD. *n* = 6; * *p* < 0.05; *** *p* < 0.001.

LOV domain was described as a reversible photochromic switch, which can be activated by blue light, and deactivated by near-UV light, or in the dark [28]. To check that this photochromic switch and the subsequent activation of the TLR4 is reversible, the opto-TLR4 PANC-1 cells were alternately illuminated and incubated in the dark, and NF-κB and ERK signalling was measured (Figure 5). More precisely, the opto-TLR4 PANC-1 were cultivated and starved for 24 h with serum-free culture medium and exposed to light at 470 nm for 7.5 min or treated with 100 ng/mL LPS and incubated for 24 h in dark before cells were again stimulated with light or LPS, respectively. NF-κB signalling was determined by measuring Gluc 6 h and 24 h after each stimulation (Figure 5A,B). As depicted in Figure 5B, NF-κB signalling already increased significantly 6 h after light exposure, peaking after 24 h due to accumulation of Gluc in the medium. Re-stimulation of LPS showed a more pronounced increase in NF-κB activity than re-illumination at 470 nm (T48h). However, NF-κB expression after the third light treatment already aligned to the NF-κB expression level induced by LPS (T72h) and, evidently, exceeded it after the fourth stimulation (T96h).

Next, cells were treated as before and protein expression of phospho-p65 and phospho-ERK1/2 was analysed 0.5 h, 3 h, 8 h, and 24 h after the first stimulation with light or LPS, respectively, and 8 h after the second stimulation (32 h). As shown in Figure 5C–G, phosphorylation of both proteins p65 and ERK1/2 could be turned on by light and off in the dark, clearly demonstrating the reversibility of the photoreaction of the LOV domain and, corresponding, TLR4 signalling pathways (Figure 5C,D). When cells were exposed for 7.5 min, p65 and ERK1/2 phosphorylation increased already after 0.5 h, peaking at 8 h for p65 and 0.5 h for ERK1/2, and reached the ground-level 24 h post illumination. In comparison, continuous stimulation with light or LPS indicated strong p65 and ERK 1/2 phosphorylation even after 24 h (Figure 5E–G).

### 2.4. Light Induced TLR4 Activation Did Not Change Cell Viability, but Increased Cell Adhesion to, and Invasion into Extracellular Matrix

**Figure 6:**
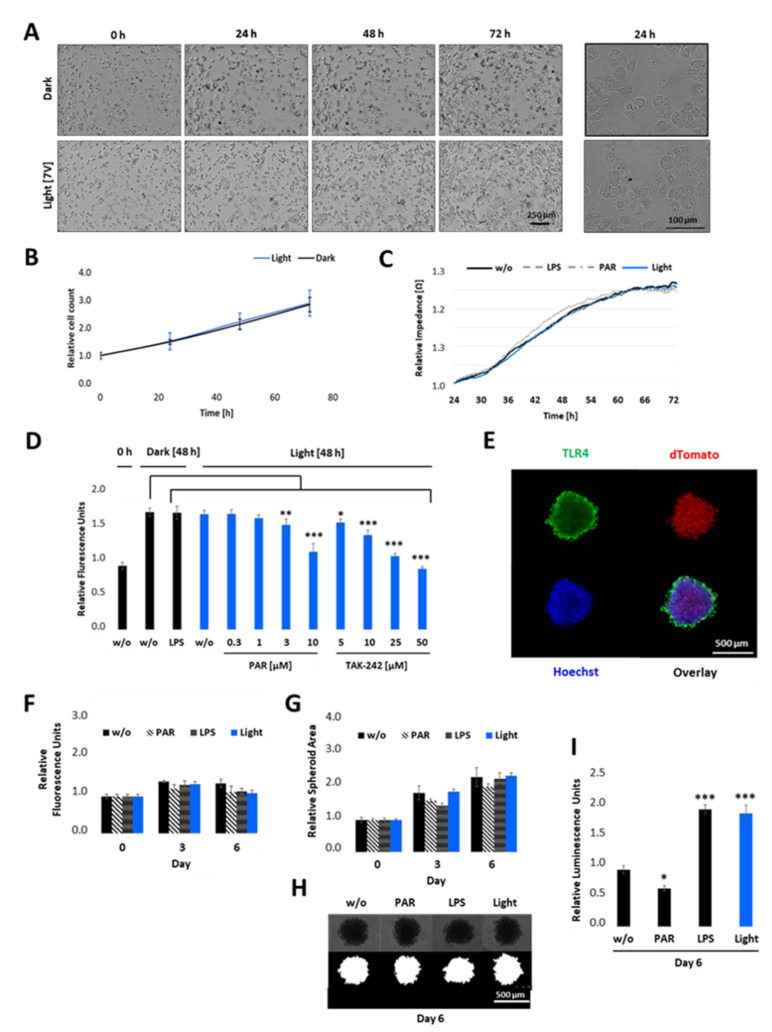
Cell proliferation and viability of opto-TLR4 PANC-1 upon illumination in 2D cultures and 3D spheroids. (**A**,**B**) Opto-TLR4 PANC-1 cell line (3000 cells/ well plate) were either exposed to light for 72 h or kept in the dark. Phase contrast images were taken (**A**) and cell count **(B)** was measured every 24 h using the MiniMaxTM 300 Imaging Cytometer. (**C**) Cells were allowed to attach on ECIS array and 24 h later they were exposed to light, treated with 100 ng/mL LPS or 3 µM PAR, or left untreated for 48 h and changes in impedance measured in real-time. (**D**) Cells were non-treated, treated with 100 ng/mL LPS, or exposed to light (470 nm) +/− different concentrations of PAR or TAK-242 and cell viability measured 48 h later using PrestoBlue assay. (**E**) 2000 opto-TLR4 PANC-1 cells were cultured for 4 days as three-dimensional (3D) cell aggregates (spheroids) using non-adherent plates and processed for immunofluorescence microscopy using TLR4 antibody and Hoechst dye. Images were acquired (TLR4-Alexa-488: green, dTomato: red, Hoechst: blue) using a Leica TCS SP8 confocal laser scanning microscope. 5× magnification. Scale bars: 500 μm. (**F**–**I**) Spheroids were treated with 100 ng/mL LPS or 3 µM PAR, exposed to light (470 nm) for 7.5 min twice a day, or left untreated for 6 days and (**F**) cell viability was measured using PrestoBlue assay. (**G**) Spheroid area was measured on days 0, 3, and 6 by segmenting bright field images (**H**) using the FIJI Macro INSIDIA. Data were collected at least 3 times in octuplicates and normalised to the non-treated control. (**I**) Relative NF-κB promoter-linked Gaussia Luciferase activity in opto-TLR4 PANC-1 spheroids on day 6. Post-ANOVA multiple comparisons relative to the control were performed using Dunnett’s test. Error bar = SD. *n* = 6; * *p* < 0.05; ** *p* < 0.01, *** *p* < 0.001.

Since TLR4-mediated signalling has been reported to be responsible for cell survival, cancer progression, invasion, and metastasis [9,10,12], we next developed phenotypic assays in order to better understand the complex interplay of tumour progression and the TLR4 signalling pathway. First, we studied the role of light-induced TLR4 signalling in cell proliferation and survival in 2D cultures. Therefore, opto-TLR4 PANC-1 cells were allowed to attach to collagen type I coated wells for 24 h before cells were exposed to light, left in the dark, or were stimulated with LPS or PAR. Then, cell proliferation/viability was measured. As such, we observed that neither light exposure nor treatment with LPS or PAR influenced cell viability or proliferation significantly, as detected by light microscopy (Figure 6A) or monitored in real time by means of measuring the changes in impedance over time (Figure 6B). Next, cells were stimulated with LPS, left in the dark, or were exposed to light with additional PAR and TAK-242 treatment in different concentrations for 48 h, and cell viability was measured using PrestoBlue assay. As depicted in Figure 6C, light exposure or LPS treatment did not alter cell viability as long they were not treated with high PAR (3 or 10 µM) or TAK-242 (10–50 µM) concentrations.

To verify the influence of light-induced TLR4 on cell viability in a physiologically more relevant test system that better reproduces the three-dimensional (3D) complexity and growth behaviour of pancreatic carcinoma than the 2D cultures, we cultivated the opto-TLR4 PANC 1 cell line (2000 cells/well) as 3D spheroids, with an initial diameter of approximately 500 µm (Figure 6D). After four days, spheroids were treated with PAR, LPS, or exposed to light for a further six days, and cell viability, as well as size of spheroids, were measured on days 0, 3, and 6. As depicted in Figure 6E–G, no significant difference in cell viability/metabolism and spheroid size (area) between treated (LPS or PAR), light-exposed, and non-treated spheroids could be detected on days 3 or 6 post treatment. Interestingly, both LPS-stimulated and light exposed spheroids (7.5 min twice a day) still indicated a significant increase in Gluc activity, whereas PAR treatment decreased the reporter activity compared to non-treated spheroids (Figure 6H).

**Figure 7:**
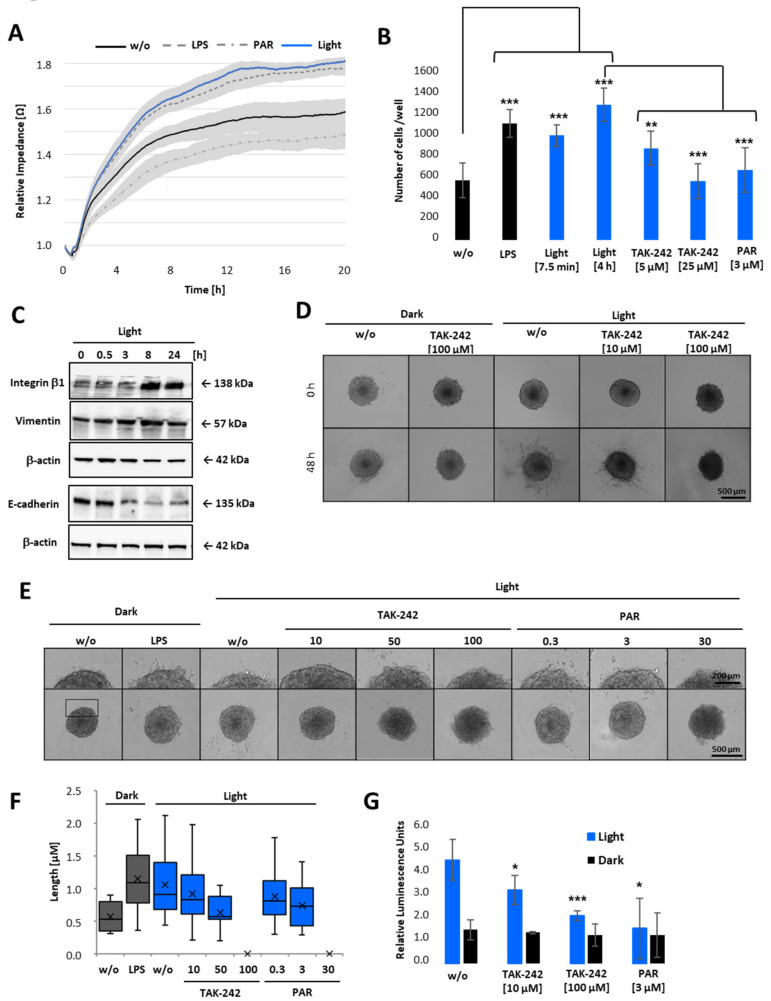
Cell attachment and 3D invasion assay of opto-TLR4 PANC-1 spheroids after light induction. (**A**) Opto-TLR4 PANC-1 cells (30,000/well) were transferred to non-adhering plates and exposed to light at 470 nm or treated with 100 ng/mL LPS, 3µM PAR, or left untreated for 6 h and seeded onto ECIS array (pre-coated with Col. I) and allowed to attach and spread for a further 20 h. (**B**) Cells were non-treated, treated with LPS, or exposed to light for 7.5 min (+6 h in the dark) or continuously for 6 h and treated with different concentrations of TAK-242 or PAR in non-adhering plates and seeded on pre-coated collagen I plates for 30 min, washed and remaining cells counted. (**C**) Western blot analysis of whole-cell extracts with antibodies against integrin β-1, vimentin, and E-cadherin after light exposure (0–24 h). (**D**) Spheroids (4 days old; 2000 cells) were embedded in Geltrex/collagen type I matrices and pre-treated 1 h with different concentrations of TAK-242 or PAR and exposed to light, treated with 1 µg/mL LPS, or left untreated and phase-contrast images taken after 0 and 48 h (**E**) or 12 h after treatment. Invadopodia extensions in 3D spheroids after 12 h light exposure are presented in corresponding images (**D**,**E**) and as a box-whisker-blot performed at least 3-times in triplicates (**F**). Scale bars: phase-contrast, (10× magnification) indicates (**D**) 500 µm or (**E**) 200 µm. (**G**) NF-κB promoter-linked Gluc detection after 12 h of light exposure. Post-ANOVA multiple comparisons relative to the control were performed using Dunnett’s test. Error bar = SD. *n* = 6; * *p* < 0.05; ** *p* < 0.01, *** *p* < 0.001.

Having demonstrated that cell viability is only decreased when cells were treated with high concentrations of PAR or TAK-242 (Figure 6C), but not due to LPS or light-induced TLR4 activation (Figure 6), we next examined whether TLR4 signalling triggers cell attachment to extracellular matrix (ECM), and/or invasion through ECM. Therefore, opto-TLR4 PANC 1 cells were left untreated (w/o), pre-treated with LPS or PAR, or illuminated with light for 6 h, and attachment and spreading on collagen type I coated wells detected in real-time for further 20 h (Figure 7A). Both LPS stimulation and light-induction promoted cell attachment and spreading compared to non-treated cells, whereas PAR stimulation showed the opposite effect. To verify this data, cells were collected in non-adhering plates and were left untreated (dark), treated with LPS, or exposed to light for 7.5 min or 6 h with 5 µM or 25 µM TAK-242 or 3 µM PAR and plated on collagen type I coated wells 6 h later. As depicted in Figure 7B, LPS treatment and exposure to light (7.5 min or 6 h) significantly increased cell attachment after 30 min, whereas the TLR4/NF-κB inhibitors TAK-242 and PAR significantly decreased this effect. Since markers such as integrin β-1 or vimentin play an essential role in adhesion and invasiveness of pancreatic carcinoma cells [29], we analysed the relationship between light-induced TLR4 activation and integrin β-1 and vimentin expression. Opto-TLR4 PANC 1 cells were illuminated with light for 0–24 h and protein expression of integrin β-1, vimentin, E-cadherin, and β-actin (loading control) was analysed using western blotting. As shown in Figure 7C, light induction increased expression of integrin β-1 after 3 h significantly and that of vimentin slightly after 8 h. In comparison, E-cadherin expression was clearly diminished after 3 h and remained downregulated even after 24 h.

After demonstrating that light-induced activation of TLR4 signalling pathway promotes cell adhesion and the expression of proteins involved in tumour promotion and metastasis, we next examined whether TLR4 activation via light exposure also promotes tumour cell invasion through ECM. Therefore, spheroids of opto-TLR4 PANC 1 cells were generated and embedded in Geltrex/collagen type I matrices for 1 h. Subsequently, cells were stimulated with different concentrations of TAK-242 or PAR and exposed to light for 48 h (Figure 7D) or 12 h (Figure 7E). Consistent with the attachment assay (Figure 7A,B), cell migration out of the spheroids and invasion into the ECM were enhanced when spheroids were LPS-treated or illuminated with light, and reduced when additionally treated with 10 or 50 µM TAK-242 and 0.3 or 3 µM PAR, respectively. High concentrations such as 100 µM TAK-242 or 30 µM PAR completely inhibited cell invasion (Figure 7D–F). As expected, also Gluc activity was increased in opto-TLR4 PANC 1 spheroids embedded in Geltrex/collagen type I matrices and could be decreased when treated with TAK-242 or PAR compared to non-treated spheroids.

## 3. Discussion

LPS/TLR4 is involved in different signalling pathways such as NF-κB, AP-1, and IRF3 cascades, which are important for the innate immune response, cell survival, cell migration and many more physiological responses. In various types of cancer, deregulation of TLR4 is closely associated with tumourigenesis and cancer progression. The pro-carcinogenic activity of TLR4 in pancreatic carcinoma is still unclear [4]. Therefore, cell cultures that allow a temporal and spatial regulation of TLR4 and the underlying signalling pathways are a beneficial tool for research and drug development.

Hence, we set out to develop a novel optogenetic cell line that enables time-resolved activation of NF-κB signalling pathway upon blue light-sensitive homodimerisation of the TLR4-LOV fusion protein. The designed reporter construct NF-κB-TRE-Gluc, CMV-dTomato allows a simultaneous expression of the reporter genes dTomato and NF-κB-Gluc. The constitutive dTomato expression enables spatial detection of the cells, while the Gluc assay permits a quantitative and time-resolved monitoring of NF-κB activity in response to inflammatory stimuli. For the construction of the TLR4-LOV plasmid, the photosensitive protein domain (LOV) aureochrome-1, isolated from the Vaucheria frigida, was fused to the C-terminal end of the full-length human TLR4. *Aureochrome-1* (AUREO1) has been described to function as a blue light-regulated transcription factor that has a LOV domain in the C-terminal region of a bZIP domain, an α-helical DNA-binding motif responsible for blue light-induced branching and for the development of a sex organ, respectively. Blue light activation of AUREO1, which exists as a monomer in reducing conditions, induces dimerisation, which subsequently increases affinity for the target DNA sequence [25,26]. By screening different LOV-sensing domains, Grusch et al. [15] clearly demonstrated that the domain found in AUREO1, in particular, resulted in homodimerisation and activation after low-intensity blue light exposure, when fused to the C-terminal end of receptor tyrosine kinases RTKs [15,27].

Transient co-transfection of either TLR4 or TLR4-LOV with the NF-κB-TRE-Gluc reporter into HeLa, 293Ta, and PANC-1 proved the functionality of the engineered constructs, as light exposure induced high NF-κB activation in cells transfected with TLR4-LOV, but not with TLR4. In contrast, LPS stimulation activated NF-κB activity in both, TLR4 and TLR4-LOV transfected cell lines.

Using the lentiviral delivery system, the NF-κB-TRE-Gluc reporter was first stably integrated into human PANC-1 cells. The obtained PANC-1 NF-κB reporter clone allows for both, up- and downregulation of the NF-κB-dependent luciferase activity in a time- and dose-dependent manner after TNFα/LPS and or PAR/TAK-242 treatment, respectively.

Next, the TLR4-LOV fusion protein was stably transfected into the engineered PANC-1 NF-κB reporter cell line and demonstrated that TLR4 can be time and dose-dependent activated by blue light and turned off in the dark.

TLR4 is a type I membrane receptor primarily activated by LPS, a core component of the outer membrane of Gram-negative bacteria. The ectodomain of the TLR4 has been described to be important for both avoiding constitutive, ligand-independent receptor signalling [28], and providing ligand regulated recognition and dimerisation of TLR4. The latter causes TLR4 Toll/IL-1R (TIR) domains rearrangement, followed by the recruitment of signalling adapter proteins and activation of the corresponding signalling cascades [29,30,31]. Hence, we tested whether light-induced dimerisation via the LOV domains may also trigger the activation of the full-length receptor. We first screened stably transfected cell clones that exhibit TLR4 dependent NF-κB Gluc activation in response to blue light. The opto-TLR4 PANC-1 clone selected here allows a dose-dependent and temporally precise dimerisation and activation of the TLR4 and respective endogenous downstream signalling cascades. Furthermore, the cell line enables the TLR4 to be continuously turned on or off by light illumination or incubation in the dark, demonstrating the reversibility of the photoreaction of the LOV domain.

Additional to UV radiation, blue light has also been documented to influence cellular physiology in different cell lines [32,33]. Therefore, we tested whether blue light exposure causes cell toxicity or changes in cell behaviour. By investigating the effects of blue light on human dermal fibroblasts, Opländer et al. [34] demonstrated that blue light emitted at 410 or 420 nm showed dose-dependent toxicity and oxidative stress, while longer wavelengths (453, 480 nm) did not display any toxicity, but displayed effects on proliferation. At this point, using the specified settings, we could confirm that illumination with blue light at 470 nm (4.5 or 7 volts; 500 ms pulse width; 250 ms pulse interval; 100 µs repeat interval; 5.0 V amplitude) did not show cytotoxic effects, changes in cell morphology or cell proliferation, even when exposed continuously for more than 24 h. However, if the voltages were higher than 7 (10 V, 13,5 V) a dose-dependent decrease in cell viability could be detected, most likely due to the heating effect of the lamp.

In summary, compared to standard cell lines, the opto-TLR4 PANC-1 cell line offers additional features that are advantageous for research and drug development. The light-inducible cell line, in comparison to the ligand-inducible one, enables physiological relevant activation levels, comparable to LPS, but allows additionally a dosage, temporal, and spatial control of TLR4 activation and a real-time measurement of the NF-κB signalling via stable integrated Gaussia Luciferase. Through single or multiple light-induction or incubation of the cells in the dark, it is possible to continuously change or terminate cell signalling allowing for better understanding of the intracellular NF-κB pathway. This is not possible through receptor activation via ligands such as LPS, as instantaneous removal of the ligand requires additional procedure steps, such as cell washing, medium change, etc., that influence cell behaviour and physiology. Furthermore, ligands such as LPS are dependent on their co-receptors [29,30] but can also bind to transient receptor potential (TRP) channels that have recently been identified as non-TLR4 membrane-bound sensors of LPS [35]. Another problem with LPS is the potential induction of off-target effects caused by sample impurities or contamination with nucleic acids and proteins [36]. All these experimental uncertainties and additional processing steps can be avoided with the opto-TLR4 PANC-1 cell line, as light allows a ligand-free and specific control of the TLR4 without changing or disturbing the cellular environment.

To study the key roles of TLR4 signalling in cancer progression and metastasis, we next established different cellular models in which cell behaviour is under optical control. Using 2D cultures and free-floating 3D spheroids, we demonstrated that light-induced activation of TLR4/NF-κB has no significant effect on cell proliferation or toxicity, whereas blocking of TLR4 activity via TAK-242 or the NF-κB signalling pathway via PAR significantly inhibited cell viability and induced apoptosis, indicating that a basic expression of TLR4/NF-κB signalling is important for cell survival. Recent studies have shown that TLR4 overexpression not only counteracts apoptosis, but also promotes proliferation, invasion, and metastasis in several cancer cells [4,9,10,12,37]. In contrast, other studies have demonstrated that LPS-induced TLR4 signalling increases cell migration and adhesion to endothelial cells or ECMs, but no significant changes in apoptosis or proliferation have been documented [38]. Sun et al. [12] clearly proved that TLR4 expression was significantly enhanced in pancreatic cancer tissues and has an important role in tumorigenesis and progression of pancreatic cancer. Our results also indicated that LPS or light-induced TLR4 activation had no significant impact on cell proliferation, but the TLR4 antagonist TAK-242 was able to reduce proliferation and cell viability in a concentration-dependent manner. These results suggest that the present expression level of TLR4 in PANC-1 cell line, in addition to TLR4-independent mechanisms, triggers those signalling cascades that lead to a very high rate of proliferation in already unstimulated cells. Therefore, further stimulation remains ineffective. Nevertheless, light-induced TLR4 activation significantly increased the invasive potential and induced EMT of the opto-TLR4 PANC-1. Activation of TLR4 signalling by light illumination markedly increased cell attachment to collagen I in a time-dependent manner, whereas TLR4 or NF-κB inhibition showed the opposite effect. Additionally, activation of TLR signalling by light illumination for only 7.5 min profoundly upregulated integrin β-1 and the mesenchymal markers vimentin and decreased the expression of the epithelial marker E-cadherin. Moreover, light exposure of ECM-loaded opto-TLR4 PANC-1 spheroids enhanced invadopodia formation and cell invasion into ECM, whereas blocking of TLR4 and NF-κB signalling inhibited this effect in a concentration-dependent manner. This is consistent with a report employing pancreatic PCa cells, as well as other cancer cells, which suggested that TLR4/NF-κB activation enhances tumour invasion and metastasis [39,40,41].

Accordingly, this opto-TLR4 PANC 1 reporter cell line offers a novel tool for analysing the role of TLR4 signalling in tumour invasion and metastasis, thereby enhancing our understanding of the pathogenesis of pancreatic cells, and providing clues for developing new strategies against TLR4-mediated metastasis.

## 4. Materials and Methods

### 4.1. Cells and Cell Culture

Human pancreatic carcinoma of ductal origin (PANC-1; ATCC^®^, Manassas, VD, USA; CRL-1469™) (RRID: CVCL_0480), HeLa (ATCC^®^, Manassas, VD, USA; CCL-2™) (RRID: CVCL_0030), and 293Ta (GeneCopoeia^TM^ , Rockville, MD, USA; LT008) (RRID: CVCL_BT05) were cultivated in DMEM growth medium (Thermo Fisher Scientific, Vienna, Austria; 31053044) supplemented with 100 U/mL Penicillin/Streptomycin (Thermo Fisher Scientific, Vienna, Austria; 15140-122), 2 mM L-glutamine (Thermo Fisher Scientific, 25030-24) and 10% fetal calf serum (FCS; Thermo Fisher Scientific, Vienna, Austria; 10270-098) at 37 °C and 5% CO_2_ in a humidified atmosphere. PANC-1 with stable integrated NF-κB-Gluc reporter and stable integrated opto-TLR4-LOV construct were cultivated with 100 µg/mL Hygromycin B (Thermo Fisher Scientific, Vienna, Austria; 10687010) and 1 µg/mL puromycin dihydrochloride (Thermo Fisher Scientific, Vienna, Austria; A1113803), respectively. The cells were passaged every 3–5 days before reaching 80% confluency using 0.25% Trypsin-EDTA (Thermo Fisher Scientific, Vienna, Austria; 25200-056) for cell detachment. For the 3D cell culture, 2000 cells were grown for 4 days in 200 mL serum reduced (1% FCS) cell culture medium using PrimeSurface 96U plates (S-Bio, Hudson, NH, USA; MS-9096UZ,) to allow spheroid formation before being used for the experiments.

### 4.2. Cloning of Reporter and Opto-Tlr4-Lov Fusion Construct

The NF-κB-TRE-Gluc, Hygro reporter (THP, Vienna, Austria; CS-NF-κB-02) was designed with five tandem repeats of NF-κB transcription responsive elements (TREs; TGGGGACTTTCCGC), the Gluc, the dTomato fluorescence protein under the control of the CMV promoter (constitutive expression of dTomato) and Hygromycin B (resistance gene) as selection marker and cloning/synthesis of the DNA construct into the pEZ-Lv195 vector (GeneCopoeia^TM^, 1 Rockville, MD, USA) commissioned.

The full-length homo sapiens Toll-like receptor 4 (TLR4), transcript variant 1, was cloned / synthesised into the lentiviral vector pEZ-Lv195 (plasmid size: 9784 bp; selection marker: puromycin; GeneCopoeia^TM^, Rockville, MD, USA). The light-oxygen-voltage-sensing (LOV) domain was cloned C-terminally into the pEZ-Lv195-TLR4 plasmid) by blunt end ligation using Blunt/TA Ligase Master Mix (New England BioLabs, Frankfurt, Germany; M0367S) of the PCR products and transfected in Escherichia coli strain GCI-5α (GeneCopoeia^TM^, 1 Rockville, MD, USA; CC001) using heat shock method (42 °C for 45 sec). The mV-VfAU1-LOV_226 was kindly provided by Harald Janovjak (Addgene, Teddington, United Kingdom; plasmid 58686) [15]. Primers to amplify the LOV domain by PCR were as follows. Forward: phospho-5′-TACAAGGGCAGTTCAGGATCA-3′, and reverse: 5′-GCAACTAGAAGGCACAGTCG -3′and/or reverse_2: 5′-CTTTCTGCGCAGCATGTTA-3′. For pEZ-Lv195 (incl. TLR4) vector following primers were used. Forward: 5′-ATCTAGCTCGAGTGCGGCCG-3′ and reverse: 5′-TGCTTCCTGCCAATTGCATCC-3′. PCR was performed with Phusion High-Fidelity DNA Polymerase (New England BioLabs, Frankfurt, Germany; M0530S) according to manufacturer’s instructions. Insertion of the LOV domain was verified using PCR and Sanger sequencing. DNA sequencing was carried out by Microsynth Austria GmbH (Vienna, Austria) and aligned against the expected sequence (TLR4-LOV) using the publicly available software tool Clustal Omega from EMBL-EBI to verify its consensus. Primer sequence 1: CTCGCATCTCTCCTTCACG, Primer sequence 2: TACAGAAGCTGGTGGCTGTG, Primer sequence 3: CAACAAAGGTGGGAATGCTT, Primer sequence 4: CGGTCCTCAGTGTGCTTGTA. (Sequence: see Additional file 1: Figure S1).

### 4.3. Transfection

HeLa, 293Ta, and PANC-1 were transiently co-transfected with NF κB-TRE-Gluc, Hygro (THP, Vienna, Austria; CS-NF-κB-02) reporter plasmid and the engineered TLR4 or TLR-LOV plasmid, respectively, using Lipofectamine 2000 (Thermo Fisher Scientific, Vienna, Austria; 11668027) according to the manufacturer’s instructions.

For lentiviral transfection, NF-κB-TRE-Gluc, Hygro (THP, Vienna; Austria; CS-NF-κB-02) and opto-TLR4-LOV pEZ-Lv195 vector (GeneCopoeia^TM^, Rockville, MD, USA), respectively, were transfected into 293Ta lentiviral packaging cells (GeneCopoeia^TM^, Rockville, MD, USA; LT008) using the Lenti-Pac™ HIV Expression Packaging Kit (GeneCopoeia^TM^, Rockville, MD, USA; LT001) according to the manufacturer’s instructions, and the supernatant with viral particles, which has harvested 48 h later was used either directly, used for infection or stored at –80 °C. For transfection, 50,000/24-well plate PANC-1 cells (+/− NF-κB-Gluc) were infected with 0.5 mL of virus suspension diluted in complete cell culture medium with 8 µg/mL polybrene, medium replaced after 24 h with complete cell culture medium (10% FCS) and selected 48 h later with 100 µg/mL Hygromycin B (Thermo Fisher Scientific, Vienna, Austria; 10687010) (PANC-1 NF-κB) or 100 µg/mL Hygromycin B and 1 µg/mL puromycin dihydrochloride (Thermo Fisher Scientific, Vienna, Austria; A1113803), respectively, (opto-TLR4 PANC-1). Single cell clones were picked, cultivated, and tested for reporter activity.

### 4.4. Substance Treatment and Light Stimulation

A total of 20,000 PANC-1 NF-κB or opto-TLR4 PANC-1 cells were cultivated in 96-well plates (Sarstedt, Nümbrecht, Germany; 833924300) for 24 h (37 °C and 5% CO2) and treated with 30 ng/mL human tumour necrosis factor-α (TNF- α; Merck, Darmstadt, Germany; T6674-10UG), 0.1-1 µg/mL lipopolysaccharides from Escherichia coli O55:B5 (LPS; Merck, Darmstadt, Germany; L4524-5MG), 0.3-30 µM parthenolide (PAR; Abcam, Cambridge, United Kingdom; ab120849), 5-100 µM TAK-242 (resatorvid; Merck, Darmstadt, Germany; 614316-5MG), a combination thereof, or left untreated and incubated for 6-48 h at 37 °C and 5% CO2 in a humidified atmosphere.

For light-induced TLR-4-LOV activation, cells were exposed to blue light using the Amuza (San Diego, CA, USA) LED array system 10335, consisting of the LEDA-B LED array with 96 LED elements, and the LAD-1 LED array driver for the definition of the corresponding parameters. Parameters used were wavelength: 470 nm; time: 2.5–60 min (followed by incubation of 6–24 h in the dark) or 6–48 h (w/o incubation in the dark); voltage: 4.5–13,5 V; delay: 0 µs; pulse width: 500 ms; pulse interval: 250 ms; repeat interval: 100 µs, number of repeats:1; amplitude: 5.0 V and the mode was hold constant.

### 4.5. Gaussia Luciferase Reporter

Gaussia Luciferase Reporter (Gluc) was measured by applying the BioLux^®^ Gaussia Luciferase Assay Kit (New England Biolabs, Frankfurt, Germany; E3300L) before (T0h) and 6-, 12-, 24-, or 48-h post (T6h-48) treatment or light exposure. Therefore, 20 µL of cell culture medium from each well was transferred to Corning^®^ 96 well plates (clear bottom, white; Merck, Darmstadt, Germany; CLS3610-48EA) and combined with 50 µL of Gluc assay solution and incubated for 30 s in the dark. Relative Luminescence Units (RLU) were measured using the Spectra Maxi3x, Luminescence Glow (Lum 384; Molecular Devices, LLC, San Jose, CA, USA; 0200-7015POS) and normalized to the cell count generated with the Mini Max 300 Imaging Cytometer (Molecular Devices, LLC, San Jose, CA, USA; 5024062).

### 4.6. Quantitative Real-Time PCR

Total ribonucleic acid (RNA) was extracted using the RNeasy^®^ Mini Kit (Qiagen, Vienna, Austria; 74104). RNA was reverse transcribed using the qScriptTm cDNA Super Mix (Quantabio, Beverly, MA, USA; 84034) according to the manufacturer’s instructions. Real-time PCR was performed with TaqMan Gene Expression Master Mix and pre-designed TaqMan^®^ Gene Expression Assays with unlabelled primers and TaqMan probes (FAM dye and quencher labelled): Hs00152939_m1 TLR4 as the target gene and Hs00183533_m1 IPO3 as the endogenous control gene (Thermo Fisher Scientific, Vienna, Austria; 4331182). Reactions were run on the Quant Studio 7 Flex (Applied Biosystems, Foster City, CA, USA; QSTUDIO7FLEX). Data were analysed using QuantStudio Real-Time PCR Software v1.3 (Applied Biosystems, Foster City, CA, USA). Expression levels of the target gene TLR4 were calculated according to the comparative Cq method (2-ΔΔCT) with IPO3 as the reference gene and fold change from opto-TLR4 PANC-1 to PANC-1 NF-κB were graphically displayed. Each experiment used at least three independent batches of RNA, and each batch was tested independently at least in triplicates.

### 4.7. Western Blotting

Protein extraction was performed with lysis buffer (500 mM NaCl, 50 mM Tris-HCl, pH 7.4, 0.1% SDS, 1% NP-40 and 0.05% NaN3) using 3.6 × 105 cells from each cell line, grown in 6-well plates (Szabo Scandic, Vienna, Austria; BDL353846). Laemmli sample buffer (Bio-Rad, Vienna, Austria; 1610747) containing 10% βmercaptoethanol (Merck, Darmstadt, Germany; M7522-100ML) was added to the protein extracts and was followed by five heating-freezing cycles composed of 95 °C for 5 min and liquid nitrogen for 1 min. Protein extracts were separated by 7.5% Mini-PROTEAN TGX Precast Protein Gels (Bio-Rad, Vienna, Austria; 456-1023) and electro-blotted onto nitrocellulose membranes (Bio-Rad, Vienna, Austria; 1704155). Membranes were blocked with 1×PBS (Thermo Fisher Scientific, Vienna, Austria; 10010-023) containing 0.1% (v/v) Tween-20 (Merck, Darmstadt, Germany; P7949-100 mL) and 5% (w/v) non-fat dry milk (New England Biolabs, Frankfurt, Germany; 9999S). Primary antibodies were obtained from US Biological (Salem, MA, USA): α-TLR4, h-Toll, CD284 (042879), Abcam (Cambridge, United Kingdom): α-phosphor S536 NF-κB (ab86299), α-NF κB p65 (ab16502) α -ERK1 (phosphor T202) & ERK2 (phospho T185) (ab201015), α-ERK1 & ERK2 (ab54230) or Santa Cruz (Dallas, TX, USA): α-Vinculin (sc-73614 HRP), α-βActin (sc-4778 HRP), α-Integrin β1 (sc-374429), α-NF-κB p65 (sc-8008 HRP), -phosphor S536 NF-κB (sc-136548 HRP), BD Transduction Laboratories: α-E-cadherin (610182), Cell Signaling (Trask Lane Danvers, MA, USA): α-Vimentin (D21H3; 5741S). Secondary antibodies were purchased from Cell Signaling: Anti-rabbit IgG, HRP-linked Antibody (7074S) or Anti-mouse IgG, HRP-linked Antibody (7076S). The immunoblot was developed by applying Clarity Western ECL Substrate (Bio-Rad, Vienna, Austria; 1705060) according to the manufacturer’s instruction. Proteins were quantified via chemiluminescence imaging using the ChemiDoc MP platform (Bio-Rad, Vienna Austria; 17001402) and in silico analysed via the Image Lab 6.0.1 Software (Bio-Rad, Vienna Austria).

### 4.8. Immunostaining

Cell monolayers or spheroids were chemically fixed with 3.8% paraformaldehyde (Thermo Fisher Scientific, Vienna, Austria; 15670799) for 30 min and permeabilized with 0.2% Triton-X-100 (Merck, Darmstadt, Germany; 11332481001) for 15 min or 30 min (spheroids). After washing, monolayers and spheroids were stained with α-TLR4, h-Toll, CD284 (US Biological, Salem, MA, USA; 042879) antibody, or for nuclear localisation with α-NF-κB p65 antibody (5 µg/mL; Abcam, Cambridge, USA; ab16502) and allowed to incubate at 4 °C overnight. Cells were washed with 1× PBS three times for 5 min and incubated for one hour at room temperature with the secondary antibody (Alexa Fluor 488 goat anti-rabbit IgG (H+L); Thermo Fisher Scientific, Vienna, Austria; A11008). A Hoechst 33342 solution (Cambrex Bioscience, Walkersville, MD, USA; PA-3014) diluted 1:1000 was added for 30 min at room temperature before cells and spheroids were finally washed with PBS and mounted for immunofluorescence confocal microscopy (Leica, Wetzlar, Germany; DMI6000B) to analyse TLR4 or p65 localisation. For quantification of nuclear localisation of NF-κB, fluorescence intensity of stained p65 in the cytoplasm and the nucleus were measured using ImageJ [42] and the mean ratio [nucleus/cytoplasm] was calculated.

### 4.9. Flow Cytometric Analysis

For apoptosis/necrosis assay, 300,000 cells were isolated, collected, and resuspended in 500 µL of 1× binding buffer (Thermo Fisher Scientific, Vienna, Austria; V35113). 5 µL of Annexin V-Alexa Fluor488 (Thermo Fisher Scientific, Vienna, Austria; V13241) and 5 µL of 7-AAD (Thermo Fisher Scientific, Vienna, Austria; 00-6993-50) were added to the solution and cells incubated in the dark. After 30 min, cells were analysed using flow cytometry and CellQuest software (BD Biosciences, Heidelberg, Germany).

### 4.10. Attachment Assay and Proliferation Assay

For the adhesion assay, opto-TLR4 PANC-1 cells were transferred to non-adhering plates (30,000 cells/ well; S-Bio, Hudson, NH, USA; MS-9096UZ) and immediately treated with LPS, or light and different concentrations TAK-242 or PAR.

For continuous measurement, the “electrical cell-substrate impedance sensing” (ECIS) model 9600Z was used. Therefore, 9W20idf PET plates (ibidi, Gräfelfing, Germany; 72098), precoated with 40 µg/mL neutralised rat tail collagen type I (Thermo Fisher Scientific, Vienna, Austria; A1048301), were overlaid with the pre-treated cells, and cell attachment and spreading were assessed by continuous resistance measurements for 20 h. For endpoint measurement, cells were plated and allowed to attach on 96-well cell culture plates (pre-coated with collagen I) for 30 min (attachment assay), rinsed twice with 1xPBS and adhering cells were automatically counted using the Mini Max 300 Imaging Cytometer (Molecular Devices, LLC, San Jose, CA, USA; 5022671).

For the proliferation assay, cells (3000 cells/well) were treated with light or left untreated for 0–72 h and cells were automatically counted using the Mini Max 300 Imaging Cytometer (Molecular Devices, LLC, San Jose, CA, USA; 5022671) every 24 h.

### 4.11. Cell Growth and Viability Assay

To measure cell growth/viability, opto-TLR4 PANC-1 cells were plated on 96-well plates for 24 h and were treated with different concentrations of PAR (Abcam, Cambridge, United Kingdom; ab120849), TAK-242 (Merck, Darmstadt, Germany; 614316-5MG) and/or illuminated with blue light for 48 h. Cell growth was detected in real-time by continuous resistance measurements for 48 h using the ECIS device (see above) or with PrestoBlue assay (Thermo Fisher Scientific, Vienna, Austria; A13262 see below) using the Spectra Max i3x Multiplate Reader and Transmitted Light (TL) detection cartridge (Molecular Devices, LLC, San Jose, CA, USA; 5022671). PrestoBlue was added to the wells to obtain a 1:10 dilution, incubated at 37 °C for 30–60 min and Ex/Em of 555/585 was measured using the SpectraMax i3x Multiplate Reader.

### 4.12. 3D Area Measurement and Invadopodia Formation

Area measurements were performed using the MiniMax 300 Imaging Cytometer (Molecular Devices, LLC, San Jose, CA, USA; 5022671). Images of the spheroids were acquired before and after treatment to define the change in size. FIJI [43] and the FIJI macro INSIDIA [44] were used to achieve spheroid segmentation and to measure the area of the segmented spheroid images, as described previously [25]. The change of each individual spheroid, relative to the same spheroid before treatment (day 0), was calculated. Furthermore, the change in the area, relative to the geometric mean of the controls without treatment within the same experiment, was calculated on days 0, 3, 6 and 9. For the 3D invasion assay, 2000 opto-TLR4 PANC-1 cells were cultivated to spheroids in 200 µL serum reduced (1% FCS) cell culture medium using Prime Surface 96U plates (S-Bio, Hudson, NH, USA; MS-9096UZ) and incubated for 4 days at 37 °C and 5% CO_2_ in a humidified atmosphere. The spheroids were embedded in extracellular matrix (ECM) consisting of equal amounts of growth medium, neutralised rat tail collagen type I (Thermo Fisher Scientific, Vienna, Austria; A1048301), and Geltrex (Thermo Fisher Scientific, Vienna, Austria; A1569601). Phase-contrast microscopy of spheroids +/− invadopodia at timepoint 0 (T0), 12 or 48 h after treatment (LPS, PAR, TAK-242, or light exposure at 470 nm for 12 h or 48 h) was performed using a Leica DMI6000B (Wetzlar, Germany) inverted microscope equipped with CTR6500 microscope drive control, DFC420C digital microscope camera with a 5 Megapixel CCD sensor and Leica Application Suite Version 3.8.0 software (Leica, Wetzlar, Germany). The number and length of invadopodia were calculated and graphically displayed using boxplot analysis.

### 4.13. Statistical Analysis

The data analysis for this paper was generated using the Real Statistics Resource Pack software (Release 7.6). Copyright (2013–2021) Charles Zaiontz. www.real-statistics.com (accessed on 11 June 2021). Post-ANOVA multiple comparisons relative to the control were performed using Dunnett’s test.

## Data Availability

Data presented in this study are available on request from the corresponding authors.

## Supplementary Materials

The following are available online at https://www.mdpi.com/article/10.3390/ijms22179232/s1.
