# Supplementary material for: Light-Inducible Spatio-Temporal Control of TLR4 and NF-κB-Gluc Reporter in Human Pancreatic Cell Line
Source: Int J Mol Sci. 2021 Aug 26;22(17):9232. doi: 10.3390/ijms22179232 (PMC8431472; doi:10.3390/ijms22179232)
Supplement: Supplementary file 1 [file ijms-22-09232-s001.zip › ijms-1317073-supplementary.pdf]

```
CLUSTAL O(1.2.4) multiple sequence alignment

GGAAAGTCCCCAGGCTCCCCAGCAGGCAGAAGTATGCAAAGCATGCATCTCAATTAGTCA      60
------------------------------------------------------------      0


GCAACCAGGTGTGGAAAGTCCCCAGGCTCCCCAGCAGGCAGAAGTATGCAAAGCATGCAT      120
------------------------------------------------------------      0


CTCAATTAGTCAGCAACCATAGTCCCGCCCCTAACTCCGCCCATCCCGCCCCTAACTCCG      180
------------------------------------------------------------      0


CCCAGTTCCGCCCATTCTCCGCCCCATGGCTGACTAATTTTTTTTATTTATGCAGAGGCC      240
------------------------------------------------------------      0


GAGGCCGCCTCTGCCTCTGAGCTATTCCAGAAGTAGTGAGGAGGCTTTTTTGGAGGCCTA      300
------------------------------------------------------------      0


GGCTTTTGCAAAAAGCTCCCGGGAGCTTGTATATCCATTTTCGGATCTGATCGGCGCGCC      360
------------------------------------------------------------      0


ATACCGGTCGCCACCATGACCGAGTACAAGCCCACGGTGCGCCTCGCCACCCGCGACGAC      420
------------------------------------------------------------      0


GTCCCCCGGGCCGTACGCACCCTCGCCGCCGCGTTCGCCGACTACCCCGCCACGCGCCAC      480
------------------------------------------------------------      0


ACCGTCGACCCGGACCGCCACATCGAGCGGGTCACCGAGCTGCAAGAACTCTTCCTCACG      540
------------------------------------------------------------      0


CGCGTCGGGCTCGACATCGGCAAGGTGTGGGTCGCGGACGACGGCGCCGCGGTGGCGGTC      600
------------------------------------------------------------      0


TGGACCACGCCGGAGAGCGTCGAAGCGGGGGCGGTGTTCGCCGAGATCGGCCCGCGCATG      660
------------------------------------------------------------      0


GCCGAGTTGAGCGGTTCCCGGCTGGCCGCGCAGCAACAGATGGAAGGCCTCCTGGCGCCG      720
------------------------------------------------------------      0


CACCGGCCCAAGGAGCCCGCGTGGTTCCTGGCCACCGTCGGCGTCTCGCCCGACCACCAG      780
------------------------------------------------------------      0


GGCAAGGGTCTGGGCAGCGCCGTCGTGCTCCCCGGAGTGGAGGCGGCCGAGCGCGCCGGG      840
------------------------------------------------------------      0

GTGCCCGCCTTCCTGGAGACCTCCGCGCCCCGCAACCTCCCCTTCTACGAGCGGCTCGGC      900
------------------------------------------------------------      0

TTCACCGTCACCGCCGACGTCGAGGTGCCCGAAGGACCGCGCACCTGGTGCATGACCCGC      960
```

```
------------------------------------------------------------    0

AAGCCCGGTGCCTAGACGCGTCTGGAACAATCAACCTCTGGATTACAAAATTTGTGAAAG    1020
------------------------------------------------------------    0

ATTGACTGGTATTCTTAACTATGTTGCTCCTTTTACGCTATGTGGATACGCTGCTTTAAT    1080
------------------------------------------------------------    0

GCCTTTGTATCATGCTATTGCTTCCCGTATGGCTTTCATTTTCTCCTCCTTGTATAAATC    1140
------------------------------------------------------------    0

CTGGTTGCTGTCTCTTTATGAGGAGTTGTGGCCCGTTGTCAGGCAACGTGGCGTGGTGTG    1200
------------------------------------------------------------    0

CACTGTGTTTGCTGACGCAACCCCCACTGGTTGGGGCATTGCCACCACCTGTCAGCTCCT    1260
------------------------------------------------------------    0

TTCCGGGACTTTCGCTTTCCCCCTCCCTATTGCCACGGCGGAACTCATCGCCGCCTGCCT    1320
------------------------------------------------------------    0

TGCCCGCTGCTGGACAGGGGCTCGGCTGTTGGGCACTGACAATTCCGTGGTGTTGTCGGG    1380
------------------------------------------------------------    0

GAAGCTGACGTCCTTTCCATGGCTGCTCGCCTGTGTTGCCACCTGGATTCTGCGCGGGAC    1440
------------------------------------------------------------    0

GTCCTTCTGCTACGTCCCTTCGGCCCTCAATCCAGCGGACCTTCCTTCCCGCGGCCTGCT    1500
------------------------------------------------------------    0

GCCGGCTCTGCGGCCTCTTCCGCGTCTTCGCCTTCGCCCTCAGACGAGTCGGATCTCCCT    1560
------------------------------------------------------------    0

TTGGGCCGCCTCCCCGCCTGGAATTAATTCTGCAGTCGAGACCTAGAAAAACATGGAGCA    1620
------------------------------------------------------------    0

ATCACAAGTAGCAATACAGCAGCTACCAATGCTGATTGTGCCTGGCTAGAAGCACAAGAG    1680
------------------------------------------------------------    0

GAGGAGGAGGTGGGTTTTTCCAGTCACACCTCAGGACCTTTAAGACCAATGACTTACAAG    1740
------------------------------------------------------------    0

GCAGCTGTAGATCTTAGCCACTTTTTAAAAGAAAAGAGGGGACTGGAAGGGCTAATTCAC    1800
------------------------------------------------------------    0

TCCCAACGAAGACAAGATCTGCTTTTTGCCTGTACTGGGTCTCTCTGGTTAGACCAGATC    1860
------------------------------------------------------------    0

TGAGCCTGGGAGCTCTCTGGCTAACTAGGGAACCCACTGCTTAAGCCTCAATAAAGCTTG    1920
```

-- 0

CCTTGAGTGCTTCAAGTAGTGTGTGCCCGTCTGTTGTGTGACTCTGGTAACTAGAGATCC 1980
-- 0

CTCAGACCCTTTTAGTCAGTGTGGAAAATCTCTAGCAGTAGTAGTTCATGTCATCTTATT 2040
-- 0

ATTCAGTATTTATAACTTGCAAAGAAATGAATATCAGAGAGTGAGAGGCTAGCGTTTTAC 2100
-- 0

CGTCGACCTCTAGCTAGAGCTTGGCGTAATCATGGTCATAGCTGTTTCCTGTGTGAAATT 2160
-- 0

GTTATCCGCTCACAATTCCACACAACATACGAGCCGGAAGCATAAAGTGTAAAGCCTGGG 2220
-- 0

GTGCCTAATGAGTGAGCTAACTCACATTAATTGCGTTGCGCTCACTGCCCGCTTTCCAGT 2280
-- 0

CGGGAAACCTGTCGTGCCAGCTGCATTAATGAATCGGCCAACGCGCGGGGAGAGGCGGTT 2340
-- 0

TGCGTATTGGGCGCTCTTCCGCTTCCTCGCTCACTGACTCGCTGCGCTCGGTCGTTCGGC 2400
-- 0

TGCGGCGAGCGGTATCAGCTCACTCAAAGGCGGTAATACGGTTATCCACAGAATCAGGGG 2460
-- 0

ATAACGCAGGAAAGAACATGTGAGCAAAAGGCCAGCAAAAGGCCAGGAACCGTAAAAAGG 2520
-- 0

CCGCGTTGCTGGCGTTTTTCCATAGGCTCCGCCCCCCTGACGAGCATCACAAAAATCGAC 2580
-- 0

GCTCAAGTCAGAGGTGGCGAAACCCGACAGGACTATAAAGATACCAGGCGTTTCCCCCTG 2640
-- 0

GAAGCTCCCTCGTGCGCTCTCCTGTTCCGACCCTGCCGCTTACCGGATACCTGTCCGCCT 2700
-- 0

TTCTCCCTTCGGGAAGCGTGGCGCTTTCTCATAGCTCACGCTGTAGGTATCTCAGTTCGG 2760
-- 0

TGTAGGTCGTTCGCTCCAAGCTGGGCTGTGTGCACGAACCCCCCGTTCAGCCCGACCGCT 2820
-- 0

GCGCCTTATCCGGTAACTATCGTCTTGAGTCCAACCCGGTAAGACACGACTTATCGCCAC 2880

TGGCAGCAGCCACTGGTAACAGGATTAGCAGAGCGAGGTATGTAGGCGGTGCTACAGAGT 2940
-- 0

TCTTGAAGTGGTGGCCTAACTACGGCTACACTAGAAGAACAGTATTTGGTATCTGCGCTC 3000
-- 0

TGCTGAAGCCAGTTACCTTCGGAAAAAGAGTTGGTAGCTCTTGATCCGGCAAACAAACCA 3060
-- 0

CCGCTGGTAGCGGTTTTTTTGTTTGCAAGCAGCAGATTACGCGCAGAAAAAAAGGATCTC 3120
-- 0

AAGAAGATCCTTTGATCTTTTCTACGGGGTCTGACGCTCAGTGGAACGAAAACTCACGTT 3180
-- 0

AAGGGATTTTGGTCATGAGATTATCAAAAAGGATCTTCACCTAGATCCTTTTAAATTAAA 3240
-- 0

AATGAAGTTTTAAATCAATCTAAAGTATATATGAGTAAACTTGGTCTGACAGTTACCAAT 3300
-- 0

GCTTAATCAGTGAGGCACCTATCTCAGCGATCTGTCTATTTCGTTCATCCATAGTTGCCT 3360
-- 0

GACTCCCCGTCGTGTAGATAACTACGATACGGGAGGGCTTACCATCTGGCCCCAGTGCTG 3420
-- 0

CAATGATACCGCGAGACCCACGCTCACCGGCTCCAGATTTATCAGCAATAAACCAGCCAG 3480
-- 0

CCGGAAGGGCCGAGCGCAGAAGTGGTCCTGCAACTTTATCCGCCTCCATCCAGTCTATTA 3540
-- 0

ATTGTTGCCGGGAAGCTAGAGTAAGTAGTTCGCCAGTTAATAGTTTGCGCAACGTTGTTG 3600
-- 0

CCATTGCTACAGGCATCGTGGTGTCACGCTCGTCGTTTGGTATGGCTTCATTCAGCTCCG 3660
-- 0

GTTCCCAACGATCAAGGCGAGTTACATGATCCCCCATGTTGTGCAAAAAAGCGGTTAGCT 3720
-- 0

CCTTCGGTCCTCCGATCGTTGTCAGAAGTAAGTTGGCCGCAGTGTTATCACTCATGGTTA 3780
-- 0

TGGCAGCACTGCATAATTCTCTTACTGTCATGCCATCCGTAAGATGCTTTTCTGTGACTG 3840

-- 0

GTGAGTACTCAACCAAGTCATTCTGAGAATAGTGTATGCGGCGACCGAGTTGCTCTTGCC 3900
-- 0

CGGCGTCAATACGGGATAATACCGCGCCACATAGCAGAACTTTAAAAGTGCTCATCATTG 3960
-- 0

GAAAACGTTCTTCGGGGCGAAAACTCTCAAGGATCTTACCGCTGTTGAGATCCAGTTCGA 4020
-- 0

TGTAACCCACTCGTGCACCCAACTGATCTTCAGCATCTTTTACTTTCACCAGCGTTTCTG 4080
-- 0

GGTGAGCAAAAACAGGAAGGCAAAATGCCGCAAAAAAGGGAATAAGGGCGACACGGAAAT 4140
-- 0

GTTGAATACTCATACTCTTCCTTTTTCAATATTATTGAAGCATTTATCAGGGTTATTGTC 4200
-- 0

TCATGAGCGGATACATATTTGAATGTATTTAGAAAAATAAACAAATAGGGGTTCCGCGCA 4260
-- 0

CATTTCCCCGAAAAGTGCCACCTGACGTCGACGGATCGGGAGATCAACTTGTTTATTGCA 4320
-- 0

GCTTATAATGGTTACAAATAAAGCAATAGCATCACAAATTTCACAAATAAAGCATTTTTT 4380
-- 0

TCACTGCATTCTAGTTGTGGTTTGTCCAAACTCATCAATGTATCTTATCATGTCTGGATC 4440
-- 0

AACTGGATAACTCAAGCTAACCAAAATCATCCCAAACTTCCCACCCCATACCCTATTACC 4500
-- 0

ACTGCCAATTACCCTGTGGGCGCAATTAACCCTCACTAAAGGGAACAAAAGCTGGAGCTG 4560
-- 0

CAAGCTTAATGTAGTCTTATGCAATACTCTTGTAGTCTTGCAACATGGTAACGATGAGTT 4620
-- 0

AGCAACATGCCTTACAAGGAGAGAAAAAGCACCGTGCATGCCGATTGGTGGAAGTAAGGT 4680
-- 0

GGTACGATCGTGCCTTATTAGGAAGGCAACAGACGGGTCTGACATGGATTGGACGAACCA 4740
-- 0

CTGAATTGCCGCATTGCAGAGATATTGTATTTAAGTGCCTAGCTCGATACATAAACGGGT 4800

-- 0

CTCTCTGGTTAGACCAGATCTGAGCCTGGGAGCTCTCTGGCTAACTAGGGAACCCACTGC 4860
-- 0

TTAAGCCTCAATAAAGCTTGCCTTGAGTGCTTCAAGTAGTGTGTGCCCGTCTGTTGTGTG 4920
-- 0

ACTCTGGTAACTAGAGATCCCTCAGACCCTTTTAGTCAGTGTGGAAAATCTCTAGCAGTG 4980
-- 0

GCGCCCGAACAGGGACTTGAAAGCGAAAGGGAAACCAGAGGAGCTCTCTCGACGCAGGAC 5040
-- 0

TCGGCTTGCTGAAGCGCGCACGGCAAGAGGCGAGGGGCGGCGACTGGTGAGTACGCCAAA 5100
-- 0

AATTTTGACTAGCGGAGGCTAGAAGGAGAGAGATGGGTGCGAGAGCGTCAGTATTAAGCG 5160
-- 0

GGGGAGAATTAGATCGCGATGGGAAAAAATTCGGTTAAGGCCAGGGGGAAAGAAAAAATA 5220
-- 0

TAAATTAAAACATATAGTATGGGCAAGCAGGGAGCTAGAACGATTCGCAGTTAATCCTGG 5280
-- 0

CCTGTTAGAAACATCAGAAGGCTGTAGACAAATACTGGGACAGCTACAACCATCCCTTCA 5340
-- 0

GACAGGATCAGAAGAACTTAGATCATTATATAATACAGTAGCAACCCTCTATTGTGTGCA 5400
-- 0

TCAAAGGATAGAGATAAAAGACACCAAGGAAGCTTTAGACAAGATAGAGGAAGAGCAAAA 5460
-- 0

CAAAAGTAAGACCACCGCACAGCAAGCGGCCGGCCGCTGATCTTCAGACCTGGAGGAGGA 5520
-- 0

GATATGAGGGACAATTAATTGGAGAAGTGAATTATATAAATATAAAGTAGTAAAAATTGA 5580
-- 0

ACCATTAGGAGTAGCACCCACCAAGGCAAAGAGAAGAGTGGTGCAGAGAGAAAAAAGAGC 5640
-- 0

AGTGGGAATAGGAGCTTTGTTCCTTGGGTTCTTGGGAGCAGCAGGAAGCACTATGGGCGC 5700
-- 0

AGCGTCAATGACGCTGACGGTACAGGCCAGACAATTATTGTCTGGTATAGTGCAGCAGCA 5760

-- 0

GAACAATTTGCTGAGGGCTATTGAGGCGCAACAGCATCTGTTGCAACTCACAGTCTGGGG 5820
-- 0

CATCAAGCAGCTCCAGGCAAGAATCCTGGCTGTGGAAAGATACCTAAAGGATCAACAGCT 5880
-- 0

CCTGGGGATTTGGGGTTGCTCTGGAAAACTCATTTGCACCACTGCTGTGCCTTGGAATGC 5940
-- 0

TAGTTGGAGTAATAAATCTCTGGAACAGATTTGGAATCACACGACCTGGATGGAGTGGGA 6000
-- 0

CAGAGAAATTAACAATTACACAAGCTTAATACACTCCTTAATTGAAGAATCGCAAAACCA 6060
-- 0

GCAAGAAAAGAATGAACAAGAATTATTGGAATTAGATAAATGGGCAAGTTTGTGGAATTG 6120
-- 0

GTTTAACATAACAAATTGGCTGTGGTATATAAAATTATTCATAATGATAGTAGGAGGCTT 6180
-- 0

GGTAGGTTTAAGAATAGTTTTTGCTGTACTTTCTATAGTGAATAGAGTTAGGCAGGGATA 6240
-- 0

TTCACCATTATCGTTTCAGACCCACCTCCCAACCCCGAGGGGACCCGACAGGCCCGAAGG 6300
-- 0

AATAGAAGAAGAAGGTGGAGAGAGAGACAGAGACAGATCCATTCGATTAGTGAACGGATC 6360
-- 0

TCGACGGTATCGCCTTTAAAAGAAAAGGGGGGATTGGGGGGTACAGTGCAGGGGAAAGAA 6420
-- 0

TAGTAGACATAATAGCAACAGACATACAAACTAAAGAATTACAAAAACAAATTACAAAAA 6480
-- 0

TTCAAAATTTTCGGGTTTATTACAGGGACAGCAGAGATCCAGTTTATCTAATACGACTCA 6540
-- 0

CTATAGGGAGAGAGAGAGAATTACCCTCACTAAAGGGAGGAGAAGCATGAATTCAAGGAT 6600
-- 0

CTGCGATCGCTCCGGTGCCCGTCAGTGGGCAGAGCGCACATCGCCCACAGTCCCCGAGAA 6660
-- 0

GTTGGGGGGAGGGGTCGGCAATTGAACGGGTGCCTAGAGAAGGTGGCGCGGGGTAAACTG 6720

```
------------------------------------------------------------      0

GGAAAGTGATGTCGTGTACTGGCTCCGCCTTTTTCCCGAGGGTGGGGGAGAACCGTATAT      6780
------------------------------------------------------------      0

AAGTGCAGTAGTCGCCGTGAACGTTCTTTTTCGCAACGGGTTTGCCGCCAGAACACAGCT      6840
------------------------------------------------------------      0

GAAGCTTCGAGGGGCTCGCATCTCTCCTTCACGCGCCCGCCGCCCTACCTGAGGCCGCCA      6900
------------------------------------------------------------      0

TCCACGCCGGTTGAGTCGCGTTCTGCCGCCTCCCGCCTGTGGTGCCTCCTGAACTGCGTC      6960
------------------------------------------------------------      0

CGCCGTCTAGGTAAGTTTAAAGCTCAGGTCGAGACCGGGCCTTTGTCCGGCGCTCCCTTG      7020
CGCCGTCTAGGTAAGTTTAAAGCTCAGGTCGAGACCGGGCCTTTGTCCGGCGCTCCCTTG      60
************************************************************

GAGCCTACCTAGACTCAGCCGGCTCTCCACGCTTTGCCTGACCCTGCTTGCTCAACTCTA      7080
GAGCCTACCTAGACTCAGCCGGCTCTCCACGCTTTGCCTGACCCTGCTTGCTCAACTCTA      120
************************************************************

CGTCTTTGTTTCGTTTTCTGTTCTGCGCCGTTACAGATCCAAGCTGTGACCGGCGCCTAC      7140
CGTCTTTGTTTCGTTTTCTGTTCTGCGCCGTTACAGATCCAAGCTGTGACCGGCGCCTAC      180
************************************************************

GGATCTAACAAGTTTGTACAAAAAAGCAGGCTTGGAAGGAGTTCGAACCATGATGTCTGC      7200
GGATCTAACAAGTTTGTACAAAAAAGCAGGCTTGGAAGGAGTTCGAACCATGATGTCTGC      240
************************************************************

CTCGCGCCTGGCTGGGACTCTGATCCCAGCCATGGCCTTCCTCTCCTGCGTGAGACCAGA      7260
CTCGCGCCTGGCTGGGACTCTGATCCCAGCCATGGCCTTCCTCTCCTGCGTGAGACCAGA      300
************************************************************

AAGCTGGGAGCCCTGCGTGGAGGTGGTTCCTAATATTACTTATCAATGCATGGAGCTGAA      7320
AAGCTGGGAGCCCTGCGTGGAGGTGGTTCCTAATATTACTTATCAATGCATGGAGCTGAA      360
************************************************************

TTTCTACAAAATCCCCGACAACCTCCCCTTCTCAACCAAGAACCTGGACCTGAGCTTTAA      7380
TTTCTACAAAATCCCCGACAACCTCCCCTTCTCAACCAAGAACCTGGACCTGAGCTTTAA      420
************************************************************

TCCCCTGAGGCATTTAGGCAGCTATAGCTTCTTCAGTTTCCCAGAACTGCAGGTGCTGGA      7440
TCCCCTGAGGCATTTAGGCAGCTATAGCTTCTTCAGTTTCCCAGAACTGCAGGTGCTGGA      480
************************************************************

TTTATCCAGGTGTGAAATCCAGACAATTGAAGATGGGGCATATCAGAGCCTAAGCCACCT      7500
TTTATCCAGGTGTGAAATCCAGACAATTGAAGATGGGGCATATCAGAGCCTAAGCCACCT      540
************************************************************

CTCTACCTTAATATTGACAGGAAACCCCATCCAGAGTTTAGCCCTGGGAGCCTTTTCTGG      7560
CTCTACCTTAATATTGACAGGAAACCCCATCCAGAGTTTAGCCCTGGGAGCCTTTTCTGG      600
************************************************************

ACTATCAAGTTTACAGAAGCTGGTGGCTGTGGAGACAAATCTAGCATCTCTAGAGAACTT      7620
ACTATCAAGTTTACAGAAGCTGGTGGCTGTGGAGACAAATCTAGCATCTCTAGAGAACTT      660
************************************************************
CCCCATTGGACATCTCAAAACTTTGAAAGAACTTAATGTGGCTCACAATCTTATCCAATC      7680
```

```
CCCCATTGGACATCTCAAAACTTTGAAAGAACTTAATGTGGCTCACAATCTTATCCAATC      720
************************************************************

TTTCAAATTACCTGAGTATTTTTCTAATCTGACCAATCTAGAGCACTTGGACCTTTCCAG      7740
TTTCAAATTACCTGAGTATTTTTCTAATCTGACCAATCTAGAGCACTTGGACCTTTCCAG      780
************************************************************

CAACAAGATTCAAAGTATTTATTGCACAGACTTGCGGGTTCTACATCAAATGCCCCTACT      7800
CAACAAGATTCAAAGTATTTATTGCACAGACTTGCGGGTTCTACATCAAATGCCCCTACT      840
************************************************************

CAATCTCTCTTTAGACCTGTCCCTGAACCCTATGAACTTTATCCAACCAGGTGCATTTAA      7860
CAATCTCTCTTTAGACCTGTCCCTGAACCCTATGAACTTTATCCAACCAGGTGCATTTAA      900
************************************************************

AGAAATTAGGCTTCATAAGCTGACTTTAAGAAATAATTTTGATAGTTTAAATGTAATGAA      7920
AGAAATTAGGCTTCATAAGCTGACTTTAAGAAATAATTTTGATAGTTTAAATGTAATGAA      960
************************************************************

AACTTGTATTCAAGGTCTGGCTGGTTTAGAAGTCCATCGTTTGGTTCTGGGAGAATTTAG      7980
AACTTGTATTCAAGGTCTGGCTGGTTTAGAAGTCCATCGTTTGGTTCTGGGAGAATTTAG      1020
************************************************************

AAATGAAGGAAACTTGGAAAAGTTTGACAAATCTGCTCTAGAGGGCCTGTGCAATTTGAC      8040
AAATGAAGGAAACTTGGAAAAGTTTGACAAATCTGCTCTAGAGGGCCTGTGCAATTTGAC      1080
************************************************************

CATTGAAGAATTCCGATTAGCATACTTAGACTACTACCTCGATGATATTATTGACTTATT      8100
CATTGAAGAATTCCGATTAGCATACTTAGACTACTACCTCGATGATATTATTGACTTATT      1140
************************************************************

TAATTGTTTGACAAATGTTTCTTCATTTTCCCTGGTGAGTGTGACTATTGAAAGGGTAAA      8160
TAATTGTTTGACAAATGTTTCTTCATTTTCCCTGGTGAGTGTGACTATTGAAAGGGTAAA      1200
***********************************************************

AGACTTTTCTTATAATTTCGGATGGCAACATTTAGAATTAGTTAACTGTAAATTTGGACA      8220
AGACTTTTCTTATAATTTCGGATGGCAACATTTAGAATTAGTTAACTGTAAATTTGGACA      1260
************************************************************

GTTTCCCACATTGAAACTCAAATCTCTCAAAAGGCTTACTTTCACTTCCAACAAAGGTGG      8280
GTTTCCCACATTGAAACTCAAATCTCTCAAAAGGCTTACTTTCACTTCCAACAAAGGTGG      1320
************************************************************

GAATGCTTTTTCAGAAGTTGATCTACCAAGCCTTGAGTTTCTAGATCTCAGTAGAAATGG      8340
GAATGCTTTTTCAGAAGTTGATCTACCAAGCCTTGAGTTTCTAGATCTCAGTAGAAATGG      1380
************************************************************

CTTGAGTTTCAAAGGTTGCTGTTCTCAAAGTGATTTTGGGACAACCAGCCTAAAGTATTT      8400
CTTGAGTTTCAAAGGTTGCTGTTCTCAAAGTGATTTTGGGACAACCAGCCTAAAGTATTT      1440
************************************************************

AGATCTGAGCTTCAATGGTGTTATTACCATGAGTTCAAACTTCTTGGGCTTAGAACAACT      8460
AGATCTGAGCTTCAATGGTGTTATTACCATGAGTTCAAACTTCTTGGGCTTAGAACAACT      1500
************************************************************

AGAACATCTGGATTTCCAGCATTCCAATTTGAAACAAATGAGTGAGTTTTCAGTATTCCT      8520
AGAACATCTGGATTTCCAGCATTCCAATTTGAAACAAATGAGTGAGTTTTCAGTATTCCT      1560
************************************************************

ATCACTCAGAAACCTCATTTACCTTGACATTTCTCATACTCACACCAGAGTTGCTTTCAA      8580
ATCACTCAGAAACCTCATTTACCTTGACATTTCTCATACTCACACCAGAGTTGCTTTCAA      1620
************************************************************
TGGCATCTTCAATGGCTTGTCCAGTCTCGAAGTCTTGAAAATGGCTGGCAATTCTTTCCA      8640
```

```
TGGCATCTTCAATGGCTTGTCCAGTCTCGAAGTCTTGAAAATGGCTGGCAATTCTTTCCA     1680
************************************************************

GGAAAACTTCCTTCCAGATATCTTCACAGAGCTGAGAAACTTGACCTTCCTGGACCTCTC     8700
GGAAAACTTCCTTCCAGATATCTTCACAGAGCTGAGAAACTTGACCTTCCTGGACCTCTC     1740
************************************************************

TCAGTGTCAACTGGAGCAGTTGTCTCCAACAGCATTTAACTCACTCTCCAGTCTTCAGGT     8760
TCAGTGTCAACTGGAGCAGTTGTCTCCAACAGCATTTAACTCACTCTCCAGTCTTCAGGT     1800
************************************************************

ACTAAATATGAGCCACAACAACTTCTTTTCATTGGATACGTTTCCTTATAAGTGTCTGAA     8820
ACTAAATATGAGCCACAACAACTTCTTTTCATTGGATACGTTTCCTTATAAGTGTCTGAA     1860
************************************************************

CTCCCTCCAGGTTCTTGATTACAGTCTCAATCACATAATGACTTCCAAAAAACAGGAACT     8880
CTCCCTCCAGGTTCTTGATTACAGTCTCAATCACATAATGACTTCCAAAAAACAGGAACT     1920
************************************************************

ACAGCATTTTCCAAGTAGTCTAGCTTTCTTAAATCTTACTCAGAATGACTTTGCTTGTAC     8940
ACAGCATTTTCCAAGTAGTCTAGCTTTCTTAAATCTTACTCAGAATGACTTTGCTTGTAC     1980
************************************************************

TTGTGAACACCAGAGTTTCCTGCAATGGATCAAGGACCAGAGGCAGCTCTTGGTGGAAGT     9000
TTGTGAACACCAGAGTTTCCTGCAATGGATCAAGGACCAGAGGCAGCTCTTGGTGGAAGT     2040
************************************************************

TGAACGAATGGAATGTGCAACACCTTCAGATAAGCAGGGCATGCCTGTGCTGAGTTTGAA     9060
TGAACGAATGGAATGTGCAACACCTTCAGATAAGCAGGGCATGCCTGTGCTGAGTTTGAA     2100
************************************************************

TATCACCTGTCAGATGAATAAGACCATCATTGGTGTGTCGGTCCTCAGTGTGCTTGTAGT     9120
TATCACCTGTCAGATGAATAAGACCATCATTGGTGTGTCGGTCCTCAGTGTGCTTGTAGT     2160
************************************************************

ATCTGTTGTAGCAGTTCTGGTCTATAAGTTCTATTTTCACCTGATGCTTCTTGCTGGCTG     9180
ATCTGTTGTAGCAGTTCTGGTCTATAAGTTCTATTTTCACCTGATGCTTCTTGCTGGCTG     2220
************************************************************

CATAAAGTATGGTAGAGGTGAAAACATCTATGATGCCTTTGTTATCTACTCAAGCCAGGA     9240
CATAAAGTATGGTAGAGGTGAAAACATCTATGATGCCTTTGTTATCTACTCAAGCCAGGA     2280
************************************************************

TGAGGACTGGGTAAGGAATGAGCTAGTAAAGAATTTAGAAGAAGGGGTGCCTCCATTTCA     9300
TGAGGACTGGGTAAGGAATGAGCTAGTAAAGAATTTAGAAGAAGGGGTGCCTCCATTTCA     2340
************************************************************

GCTCTGCCTTCACTACAGAGACTTTATTCCCGGTGTGGCCATTGCTGCCAACATCATCCA     9360
GCTCTGCCTTCACTACAGAGACTTTATTCCCGGTGTGGCCATTGCTGCCAACATCATCCA     2400
************************************************************

TGAAGGTTTCCATAAAAGCCGAAAGGTGATTGTTGTGGTGTCCCAGCACTTCATCCAGAG     9420
TGAAGGTTTCCATAAAAGCCGAAAGGTGATTGTTGTGGTGTCCCAGCACTTCATCCAGAG     2460
************************************************************

CCGCTGGTGTATCTTTGAATATGAGATTGCTCAGACCTGGCAGTTTCTGAGCAGTCGTGC     9480
CCGCTGGTGTATCTTTGAATATGAGATTGCTCAGACCTGGCAGTTTCTGAGCAGTCGTGC     2520
************************************************************

TGGTATCATCTTCATTGTCCTGCAGAAGGTGGAGAAGACCCTGCTCAGGCAGCAGGTGGA     9540
TGGTATCATCTTCATTGTCCTGCAGAAGGTGGAGAAGACCCTGCTCAGGCAGCAGGTGGA     2580
************************************************************
GCTGTACCGCCTTCTCAGCAGGAACACTTACCTGGAGTGGGAGGACAGTGTCCTGGGGCG     9600
```

```
GCTGTACCGCCTTCTCAGCAGGAACACTTACCTGGAGTGGGAGGACAGTGTCCTGGGGCG     2640
************************************************************

GCACATCTTCTGGAGACGACTCAGAAAAGCCCTGCTGGATGGTAAATCATGGAATCCAGA     9660
GCACATCTTCTGGAGACGACTCAGAAAAGCCCTGCTGGATGGTAAATCATGGAATCCAGA     2700
************************************************************

AGGAACAGTGGGTACAGGATGCAATTGGCAGGAAGCATACAAGGGCAGTTCAGGATCATC     9720
AGGAACAGTGGGTACAGGATGCAATTGGCAGGAAGCATACAAGGGCAGTTCAGGATCATC     2760
************************************************************

CGGTCCTGACTACAGTCTCGTGAAGGCTCTGCAAATGGCACAACAGAATTTTGTCATTAC     9780
CGGTCCTGACTACAGTCTCGTGAAGGCTCTGCAAATGGCACAACAGAATTTTGTCATTAC     2820
************************************************************

AGACGCCTCCCTCCCAGACAACCCTATCGTCTACGCCAGTAGAGGGTTTCTGACACTGAC     9840
AGACGCCTCCCTCCCAGACAACCCTATCGTCTACGCCAGTAGAGGGTTTCTGACACTGAC     2880
************************************************************

AGGCTATTCTCTCGACCAGATCCTGGGCAGGAACTGCAGGTTTCTGCAAGGGCCAGAAAC     9900
AGGCTATTCTCTCGACCAGATCCTGGGCAGGAACTGCAGGTTTCTGCAAGGGCCAGAAAC     2940
************************************************************

AGACCCAAGAGCTGTGGATAAGATCAGGAATGCCATCACCAAAGGCGTTGATACCAGTGT     9960
AGACCCAAGAGCTGTGGATAAGATCAGGAATGCCATCACCAAAGGCGTTGATACCAGTGT     3000
************************************************************

CTGTCTGCTGAATTATAGACAGGATGGCACAACCTTCTGGAATCTCTTCTTCGTGGCTGG     10020
CTGTCTGCTGAATTATAGACAGGATGGCACAACCTTCTGGAATCTCTTCTTCGTGGCTGG     3060
************************************************************

ACTCAGAGATTCTAAGGGCAATATTGTCAACTACGTCGGAGTGCAGTCAAAGGTGAGCGA     10080
ACTCAGAGATTCTAAGGGCAATATTGTCAACTACGTCGGAGTGCAGTCAAAGGTGAGCGA     3120
************************************************************

AGATTATGCCAAGCTGCTGGTCAACGAGCAGAACATTGAGTACAAAGGTGTGCGCACCAG     10140
AGATTATGCCAAGCTGCTGGTCAACGAGCAGAACATTGAGTACAAAGGTGTGCGCACCAG     3180
************************************************************

TAACATGCTGCGCAGAAAGCCCGGATAAAAGCTTGATCCGGCTGCTAACAAAGCGGATCC     10200
TAACATGCTGCGCAGAAAG-----------------------------------------     3199
*******************

GAGCTCGGTACCAAGCTTAAGTTTAAACCGCTGATCAGCCTCGACTGTGCCTTCTAGTTG     10260
------------------------------------------------------------     3199

CGACCCTGCTTGCTCAACTCT   10281
---------------------   3199
```

Figure S1: Sanger sequence of the opto-TLR4-LOV. Multiple alignment of the partially Sanger sequenced DNA plasmid of the engineered TLR4-LOV construct and the expected theoretical sequence using the publicly available software tool Clustal Omega by EMBL-EBI. The different partial sequences are highlighted as followed: sequence 1; sequence 2;sequence 3; sequence 4. The LOV domain is marked in blue.

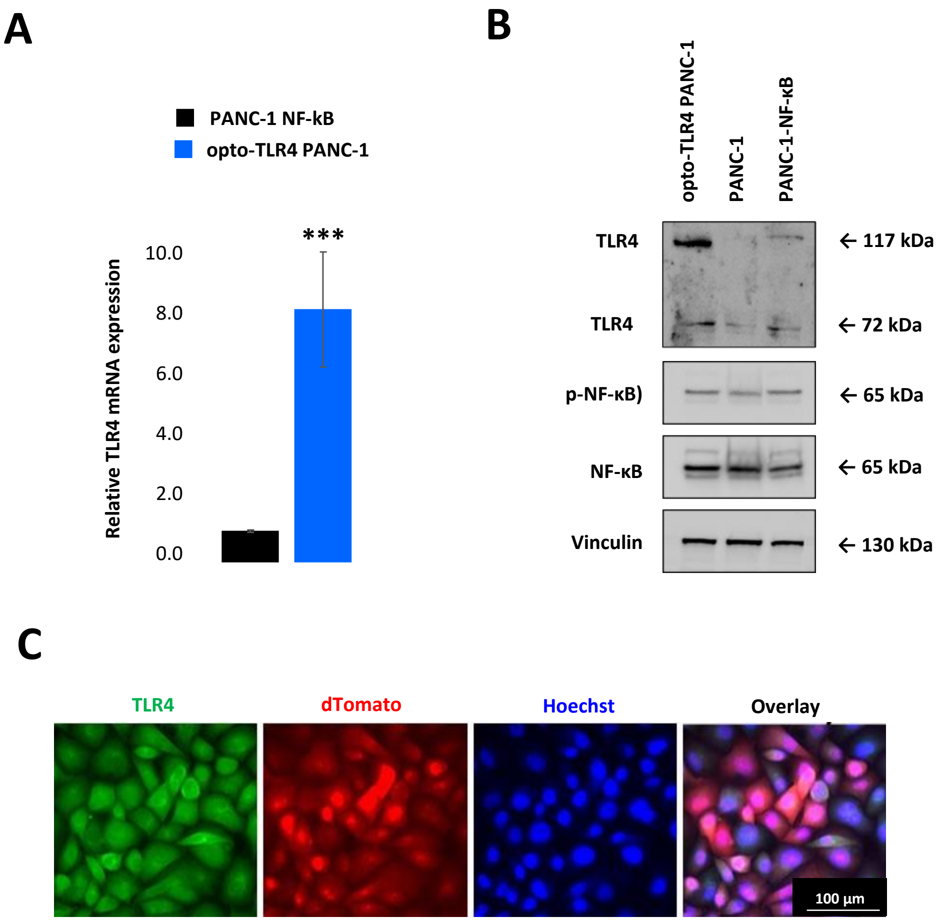

Figure S2: TLR4-LOV / NF-κB expression analysis of the engineered opto-TLR4 PANC-1 cell line. (**A**) Real-time PCR, (**B**) Western blotting and (**C**) fluorescence microscopy were performed to assess TLR4-LOV expression.
